# PRRSV promotes bacterial infection by remodeling actin cytoskeleton and cell membrane proteins

**DOI:** 10.1128/mbio.01945-25

**Published:** 2025-09-12

**Authors:** Xiao Liu, Fang Lv, Yanan Zhu, Yinan Meng, Bo Peng, Zifang Zheng, Yang Li, Lele Xu, Yingtong Feng, Jianwu Zhang, Shuqi Xiao

**Affiliations:** 1 State Key Laboratory for Animal Disease Control and Prevention, College of Veterinary Medicine, Lanzhou University, Lanzhou Veterinary Research Institute, Chinese Academy of Agricultural Sciences 12426, Lanzhou, Gansu, China; 2 College of Veterinary Medicine, Northwest A&F University 12469, Yangling, Shaanxi, China; Dana-Farber Cancer Institute, Boston, Massachusetts, USA

**Keywords:** PRRSV, secondary bacterial infection, actin cytoskeleton, ITGα5, FLNA

## Abstract

Secondary infection is a worldwide problem in the prevention and control of viral infection. Secondary bacterial infection induced by porcine reproductive and respiratory syndrome virus (PRRSV) infection causes enormous economic losses, but the relevant mechanism remains unclear. We found that the infection of *Klebsiella pneumoniae* or *Streptococcus suis type 2* in the lungs of PRRSV-challenged piglets was significantly higher than the controls, and the infection of PRRSV, influenza A virus H1N1 (H1N1), and porcine circovirus type 2 (PCV2) also significantly increased the infection of the bacteria *in vitro*. Transcriptomic analysis revealed that PRRSV infection significantly altered the expression of cytoskeleton-related proteins, among which the expression of actin-binding protein filamin A (FLNA) was significantly increased, and knockdown of FLNA could significantly reduce bacterial invasion. Mechanistic studies found that FLNA drives actin cytoskeleton rearrangement by promoting F-actin generation, thereby facilitating bacterial invasion. Further studies found that PRRSV promoted bacterial adhesion by upregulating the expression of integrin α5 (ITGα5). ITGα5 could induce actin cytoskeleton rearrangement by promoting FLNA expression, thus aggravating bacterial invasion. Furthermore, we found that lentiviral shRNA-mediated knockdown of FLNA or ITGα5 significantly reduced bacterial infection in the lungs of mice and protected mice from death. These results suggest that the regulation of actin cytoskeleton and cell membrane proteins may be a conserved mechanism of virus-induced secondary bacterial infection.

**IMPORTANCE:**

An important reason why porcine reproductive and respiratory syndrome virus (PRRSV) is difficult to control effectively is that it often causes severe secondary bacterial infections, which are usually attributed to the immunosuppression caused by PRRSV. However, the mechanism by which PRRSV infection leads to increased susceptibility of cells to bacterial infection has been largely overlooked. We revealed that PRRSV induced actin cytoskeleton rearrangement by upregulating FLNA expression, thereby aggravating bacterial invasion. PRRSV increased bacterial adhesion by promoting the ITGα5 expression, and the upregulation of ITGα5 could induce FLNA-mediated actin cytoskeleton rearrangement. Furthermore, we found that H1N1 and porcine circovirus type 2 infection also significantly promoted the expression of FLNA and ITGα5 and increased the infection of multiple bacteria. These results suggest that FLNA and ITGα5 play important roles in virus-induced secondary bacterial infection.

## INTRODUCTION

Secondary bacterial infection is the leading cause of virus-associated mortality during viral infection. The synergistic lethality of viral and bacterial coinfection has been observed in multiple viral infection processes, suggesting a causal relationship between viral infection and secondary bacterial infection (1–5). Porcine reproductive and respiratory syndrome virus (PRRSV) infection does not usually cause acute mortality in pigs, but secondary bacterial infection often results in high mortality in weaned piglets (6). In a piglet infection model, PRRSV infection significantly promoted bacterial infection and increased the mortality of piglets (7). However, the underlying mechanism of the viral-bacterial synergy that aggravates disease progression and mortality remains elusive, which hampers the generation of effective prophylactic and therapeutic options.

PRRSV has caused great harm to the pig industry worldwide (8), and the rapid mutation and frequent recombination of the virus have challenged clinical prevention and control (9, 10). As the main primary pathogen, PRRSV is prone to secondary infection with other pathogens (11), resulting in severe economic losses. Furthermore, during the severe acute respiratory syndrome coronavirus 2 (SARS-CoV-2) pandemic, secondary bacterial infection has led to severe inflammatory cytokine storm and lung tissue damage (12), increasing the difficulty of clinical treatment. This highlights the impact of secondary bacterial infections on both animal and human infections.

*Aerococcus viridans* (13), *Klebsiella pneumoniae* (14), *Streptococcus suis type 2* (15), and *Staphylococcus aureus* (16) are the major pathogens causing pneumonia in humans and animals. These bacteria rarely infect animals with a healthy immune system (17). However, the infection rate is greatly increased in the presence of other primary pathogens and underlying diseases.

This study investigated the molecular mechanisms by which respiratory viruses promote bacterial infections. The findings revealed that FLNA facilitates bacterial invasion by inducing actin cytoskeleton rearrangement, and PRRSV promotes bacterial adhesion by upregulating integrin α5 (ITGα5). Further studies showed that ITGα5 could induce actin cytoskeleton rearrangement by enhancing FLNA expression, thus promoting bacterial invasion. These findings indicate that FLNA and ITGα5 play critical roles in secondary bacterial infections following viral infection, and interventions targeting these mechanisms may be candidate options to cope with secondary bacterial infection.

## RESULTS

### PRRSV promotes bacterial infection in the lungs of piglets

**Fig 1:**
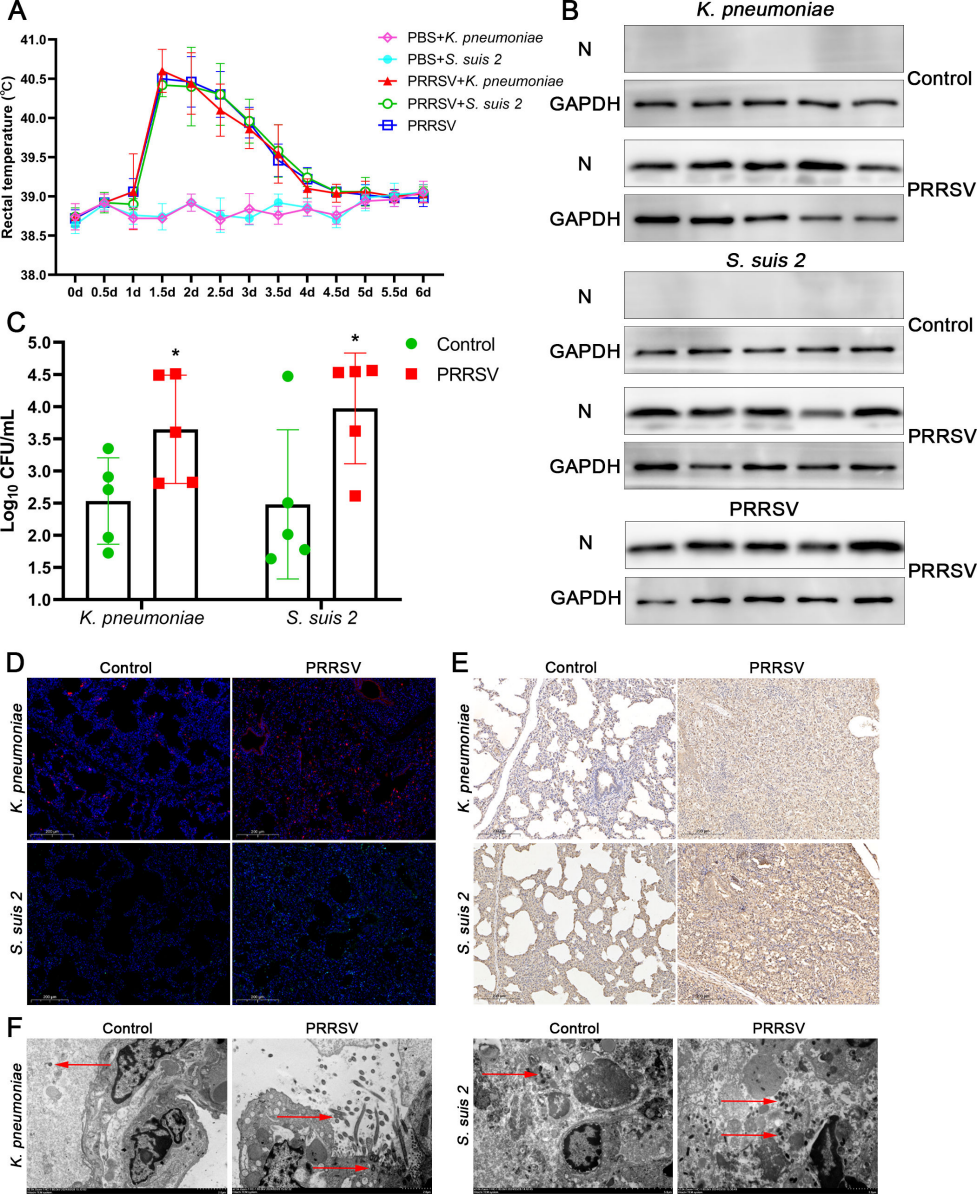
PRRSV promotes bacterial infection in the lungs of piglets. (**A**) Rectal temperature of the piglets. (**B**) After the piglets were sacrificed or died, PAMs were collected and analyzed by Western blotting. (**C**) The lung tissues of the piglets were homogenized, and bacterial invasion was quantified by CFU. (**D and E**) Lung tissue sections were prepared for immunofluorescence and immunohistochemistry detection with anti-*K*. *pneumoniae* and anti-*S*. *suis 2* antibodies. (**F**) The cells were infected with bacteria and observed by transmission electron microscopy (red arrowheads indicate bacteria).

Some studies have reported that viruses promote bacterial infection (1, 18, 19), and it has also been observed that PRRSV-positive piglets are prone to coinfection with bacteria in clinical practice (20). To investigate the effect of PRRSV on bacterial infection, piglets were infected with PRRSV or treated with phosphate buffered saline (PBS). The body temperature of the piglets increased significantly on Day 1.5 after PRRSV infection and gradually returned to normal on Day 4 (Fig. 1A). This indicates that piglets will recover from PRRSV infection, then piglets were infected with the gram-negative representative strain *K. pneumoniae* or the gram-positive representative strain *S. suis 2* on Day 5, which often cause pneumonia in piglets. Piglets in the PRRSV + bacteria groups began to die on Day 5.5 (Fig. S1A; Table 1). All piglets were sacrificed on Day 6, after which the number of bacteria in the lungs was tested. As shown in Fig. 1B, PRRSV replicated efficiently in the porcine alveolar macrophages (PAMs) of piglets, and quantification of the adherent bacteria in the lungs of piglets by CFU revealed that the numbers of *K. pneumoniae* or *S. suis 2* in the PRRSV-challenged groups were significantly greater compared with control groups (Fig. 1C). PRRSV-challenged piglets exhibited more severe pulmonary pathological lesions after bacterial infection (Fig. S1B). Severe hemorrhagic necrosis was observed in the lung tissue of piglets in the PRRSV + *K. pneumoniae* group, and more severe fibrin exudation was observed in the PRRSV + *S. suis 2* group (Fig. S1C). Immunofluorescence and immunohistochemistry were used to detect *K. pneumoniae* and *S. suis 2* infections in the lungs of piglets and revealed that the PRRSV-challenged group exhibited higher infection levels compared to the control group (Fig. 1D and E). Subsequently, transmission electron microscopy analysis of bacterial infection in piglet lung tissues revealed a significantly higher bacterial load in the PRRSV-infected group compared to the control group (Fig. 1F). Notably, although neither *K. pneumoniae* nor *S. suis 2* is classified as an obligate intracellular pathogen, both were observed to invade and localize inside cells. These results suggest that PRRSV significantly increases the infection of *K. pneumoniae* and *S. suis 2*.

**TABLE 1:** Survival statistics of piglets before sacrifice

Group	Survival	Death
PBS + *K. pneumoniae*	5	0
PRRSV + *K. pneumoniae*	3	2
PBS + *S. suis 2*	4	1
PRRSV + *S. suis 2*	2	3
PRRSV	5	0

### PRRSV infection upregulates FLNA expression

**Fig 2:**
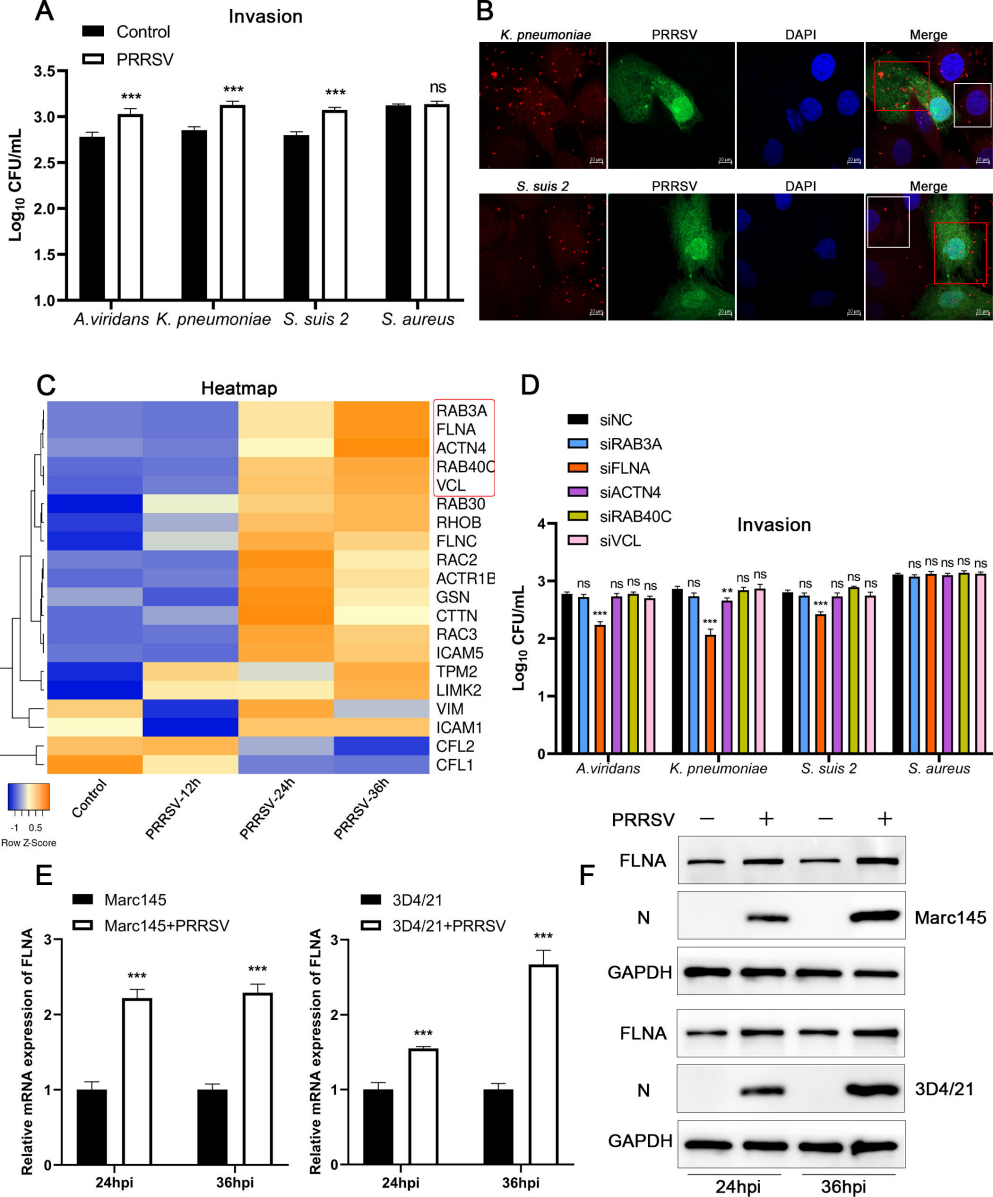
PRRSV infection upregulates FLNA expression. (**A**) PAMs were infected with PRRSV GD-HD (MOI = 1) for 24 h and then infected with *A. viridans*, *K. pneumoniae*, *S. suis 2*, or *S. aureus* (MOI = 10), and bacterial invasion was quantified by CFU. (**B**) 3D4/21 cells were infected with PRRSV GD-HD (MOI = 1) for 36 h, and then the cells were infected with *K. pneumoniae* or *S. suis 2* for 4 h. The bacterial invasion was detected by immunofluorescence. (**C**) PAMs were infected with PRRSV GD-HD (MOI = 1), and transcriptome analysis was performed at 12, 24, and 36 hpi. (**D**) Marc145 cells were transfected with VCL, RAB40C, FLNA, or ACTN4 siRNA for 24 h, then infected with *A. viridans*, *K. pneumoniae*, *S. suis 2*, or *S. aureus* (MOI = 10), bacterial invasion was quantified by CFU. (**E and F**) 3D4/21 and Marc145 cells were infected with PRRSV GD-HD (MOI = 1), then FLNA and PRRSV N expression were detected at 24 and 36 hpi.

PRRSV promotes bacterial infection, which is generally thought to be caused by immunosuppression after viral infection (21). However, the mechanism by which PRRSV infection leads to increased susceptibility of cells to bacteria remains unclear, and we found that PRRSV infection promotes bacterial invasion into cells (Fig. 1F). To explore how PRRSV increases bacterial invasion, we inoculated *A. viridans*, *K. pneumoniae*, *S. suis 2*, or *S. aureus* into PRRSV-infected or noninfected PAMs. Bacterial invasion quantified using CFU and the results revealed that PRRSV infection significantly increased the infection of *A. viridans*, *K. pneumoniae*, and *S. suis 2*, but not *S. aureus* (Fig. 2A). To further investigate the effect of PRRSV infection on bacterial invasion, we infected 3D4/21 (immortalized PAMs) cells with PRRSV for 36 h, then infected the cells with *K. pneumoniae* or *S. suis 2* and detected bacterial invasion. The results showed that the number of *K. pneumoniae* or *S. suis 2* was higher in PRRSV-infected cells (red boxes) than in uninfected cells (white boxes) (Fig. 2B). The above results indicated that PRRSV infection significantly enhanced bacterial invasion. The invasion of bacteria into cells depends on endocytosis, which requires the involvement of cytoskeleton-related proteins. Therefore, we performed transcriptomic analysis of PRRSV-infected PAMs to determine the expression changes of cytoskeleton-associated proteins following virus infection. The results showed that multiple cytoskeleton-associated proteins were significantly upregulated after PRRSV infection (Fig. 2C). Subsequently, we selected the five most prominently upregulated molecules after PRRSV infection for knockdown and examined their effects on bacterial invasion. Notably, FLNA knockdown significantly suppressed bacterial infection (Fig. 2D). We then examined the effect of PRRSV infection on FLNA expression and found that PRRSV infection significantly promoted FLNA expression in 3D4/21 and Marc145 cells (Fig. 2E and F). These results suggest that PRRSV may increase bacterial infection by promoting FLNA expression.

### FLNA knockdown significantly inhibited bacterial invasion

**Fig 3:**
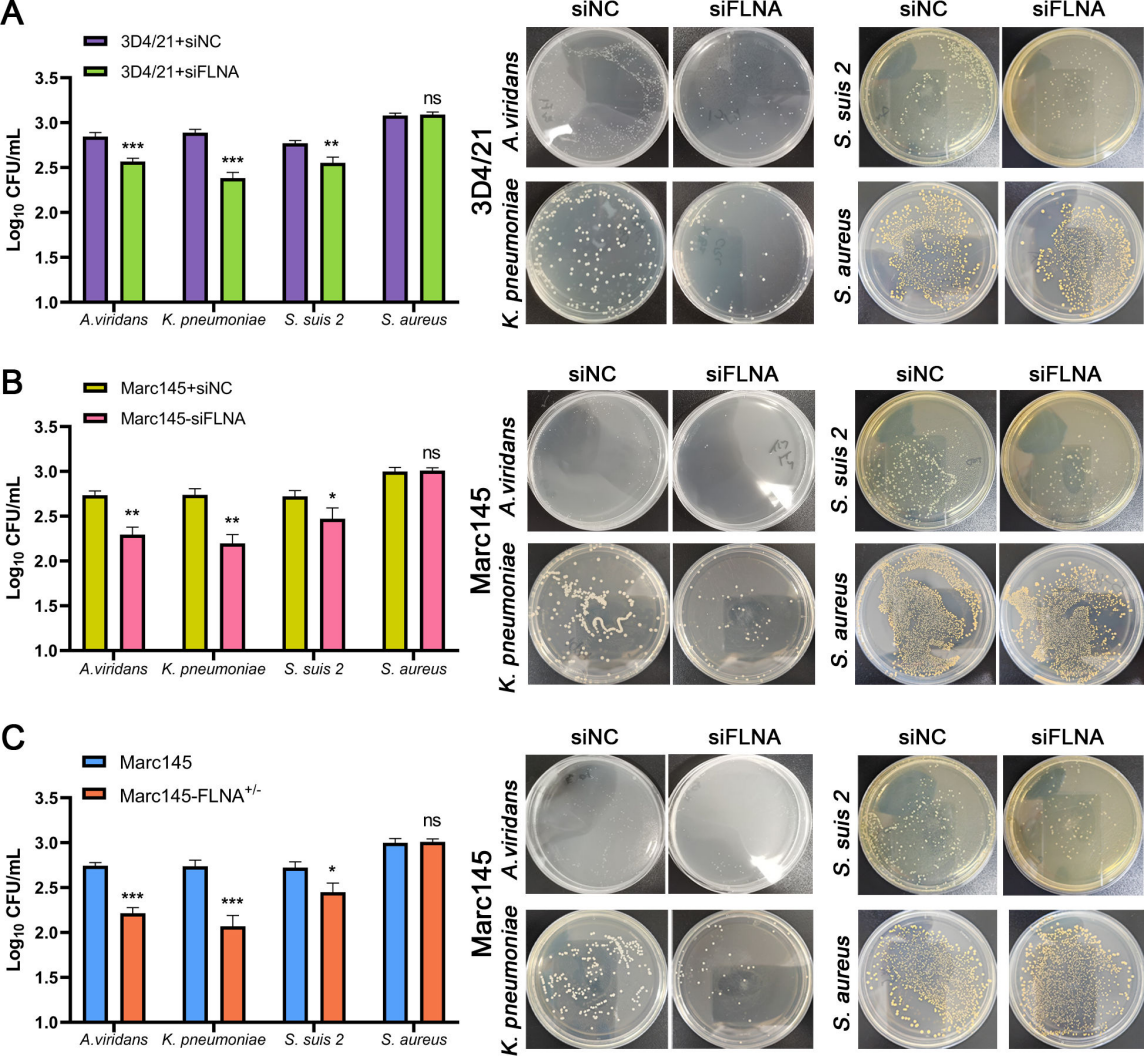
FLNA knockdown inhibited bacterial invasion. (**A and B**) 3D4/21 or Marc145 cells were transfected with FLNA siRNA for 24 h and infected with *A. viridans*, *K. pneumoniae*, *S. suis 2*, or *S. aureus* (MOI = 10), and bacterial invasion was quantified via CFU. (**C**) Marc145 and Marc145-FLNA^+/−^ cells were infected with *A. viridans*, *K. pneumoniae*, *S. suis 2*, or *S. aureus*, and bacterial invasion was quantified by CFU.

To further investigate the effect of FLNA on bacterial infection, 3D4/21 and Marc145 cells were transfected with FLNA siRNA for 24 h, then the cells were inoculated with *A. viridans*, *K. pneumoniae*, *S. suis 2*, or *S. aureus*, respectively. After infection for 4 h, bacterial invasion was tested by CFU. As shown in Fig. 3A and B, FLNA knockdown significantly inhibited the invasion of *A. viridans*, *K. pneumoniae*, and *S. suis 2*, but did not suppress that of *S. aureus*. Next, we constructed a Marc145-FLNA^+/−^ knockout cell line, infected it with bacteria, and measured bacterial invasion with CFU 4 h after infection. The results showed that the invasion of *A. viridans*, *K. pneumoniae*, and *S. suis 2* was significantly reduced in Marc145-FLNA^+/−^ cells (Fig. 3C). These results suggest that FLNA plays a key role in bacterial invasion.

**Fig 4:**
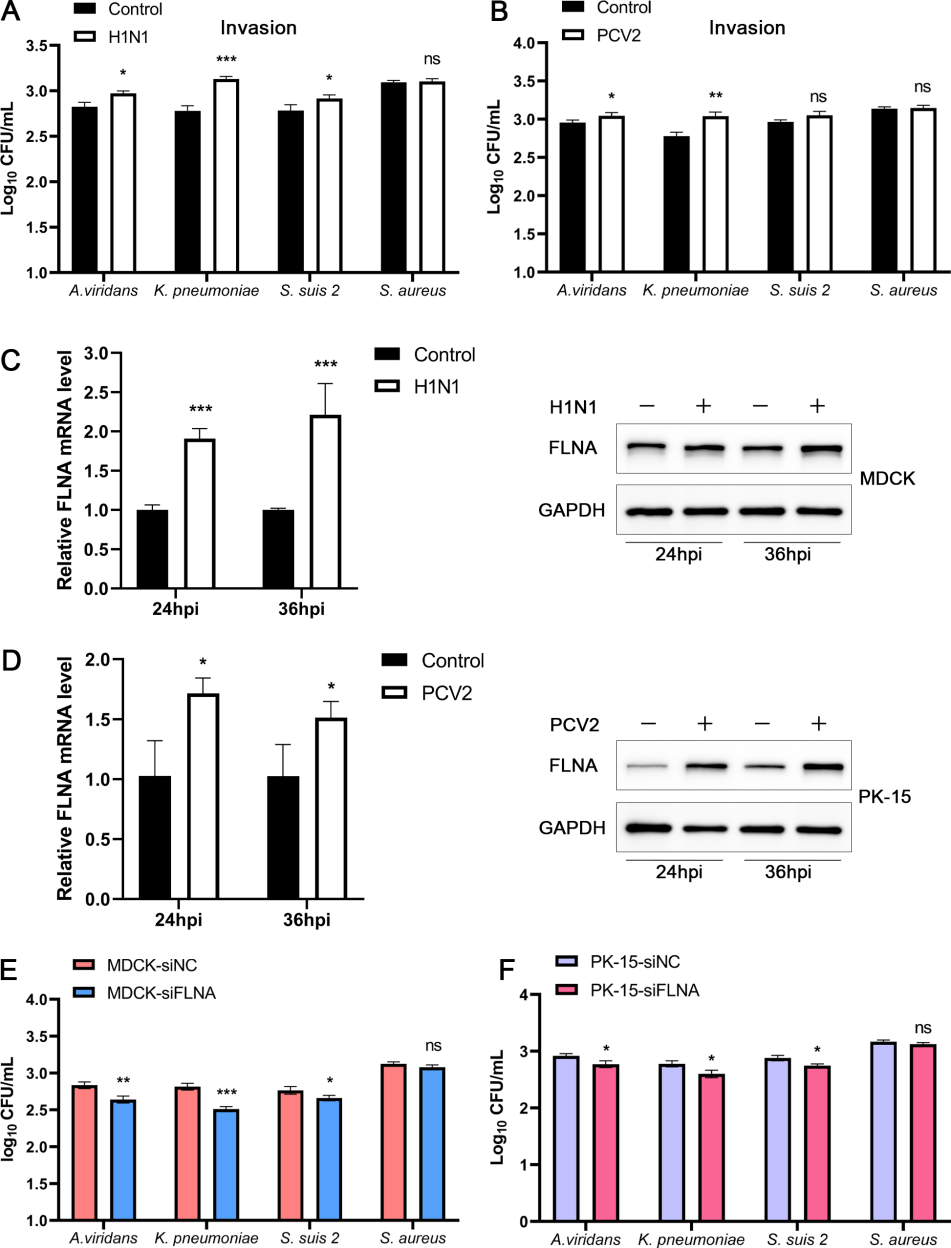
H1N1 and PCV2 infections promote FLNA expression. (**A and B**) MDCK or PK-15 cells infected with H1N1 or PCV2 (MOI = 1) for 24 h and then infected with *A. viridans*, *K. pneumoniae*, *S. suis 2*, or *S. aureus* (MOI = 10), and the number of bacterial invasion was quantified by CFU. (**C**) MDCK cells were infected with H1N1 (MOI = 1), then the expression of FLNA was detected at 24 and 36 hpi. (**D**) PK-15 cells were infected with PCV2 (MOI = 1), then the expression of FLNA was detected at 24 and 36 hpi. (**E and F**) MDCK or PK-15 cells were transfected with FLNA siRNA for 24 h and infected with *A. viridans*, *K. pneumoniae*, *S. suis 2*, or *S. aureus* (MOI = 10), and bacterial invasion was quantified by CFU.

To explore the influence of other respiratory viruses on bacterial infection, we infected cells with H1N1 or PCV2, then inoculated with *A. viridans*, *K. pneumoniae*, *S. suis 2*, and *S. aureus*, and bacterial invasion was quantified using CFU 4 h after infection. The results showed a significant increase in infections of *A. viridans* and *K. pneumoniae* in H1N1-infected and PCV2-infected cells, while there was no significant enhancement in *S. suis 2* and *S. aureus* (Fig. 4A and B). Further examination of the effects of H1N1 and PCV2 infection on FLNA expression showed that H1N1 and PCV2 infection significantly promoted FLNA expression (Fig. 4C and D). Knockdown of FLNA in MDCK and PK-15 cells, which support their proliferation, showed that FLNA knockdown significantly decreased bacterial invasion (Fig. 4E and F). These results indicate that FLNA plays a decisive role in the process of respiratory virus-induced bacterial infection.

### FLNA increases bacterial invasion by regulating F-actin production

**Fig 5:**
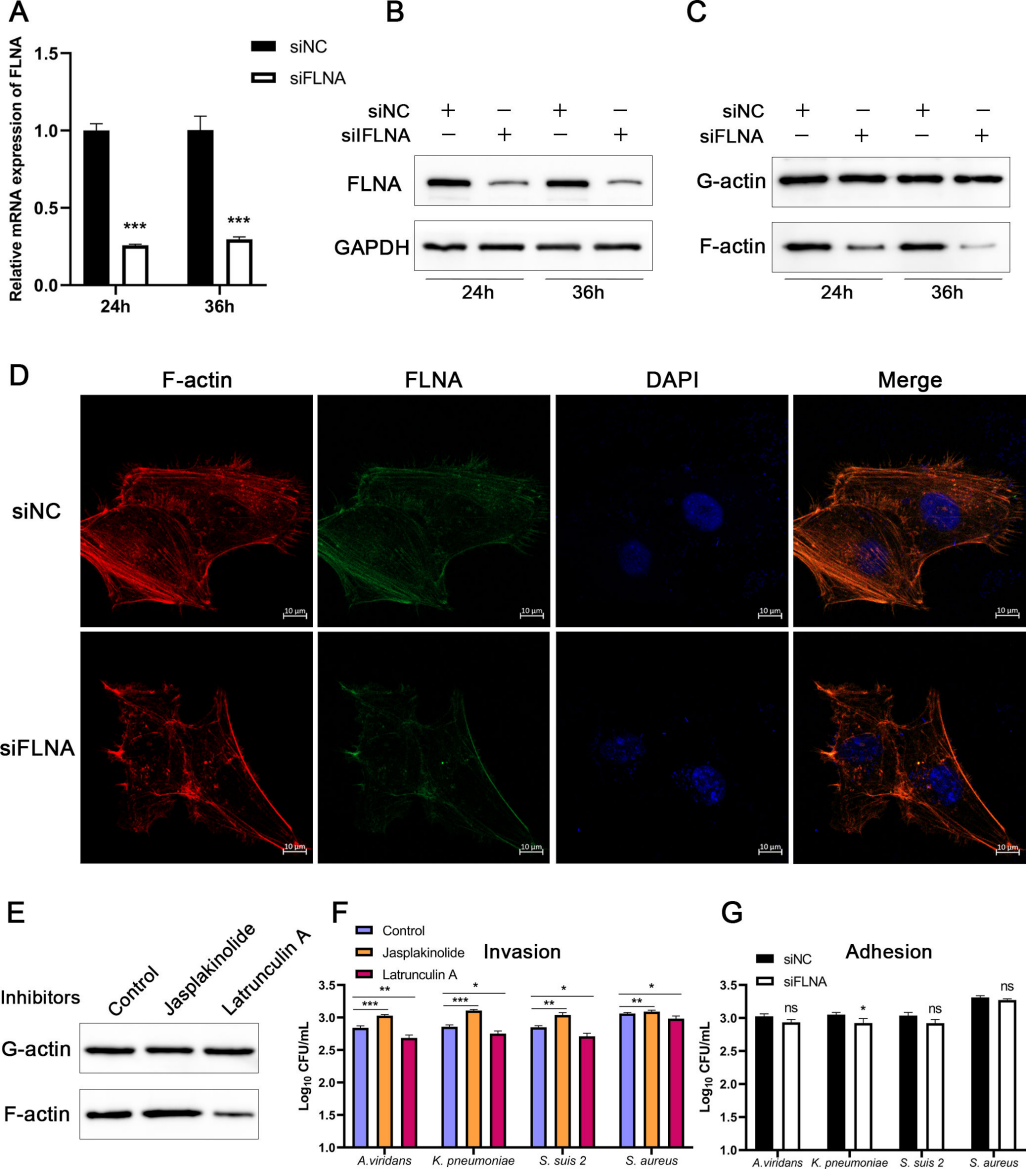
FLNA increases bacterial invasion by regulating F-actin production. (**A and B**) 3D4/21 cells were transfected with FLNA siRNA, and the mRNA and protein expression levels of FLNA were detected 24 and 36 h after transfection. (**C and D**) FLNA siRNA was transfected into 3D4/21 cells, and G/F-actin levels were detected 24 and 36 h after transfection. The cells were collected 24 h after transfection for F-actin immunofluorescence detection. (**E**) 3D4/21 cells were treated with the inhibitors Jasplakinolide or Latrunculin A for 30 or 15 min, then the cells were harvested to detect the G/F-actin protein levels. (**F**) After inhibitor treatment, the cells were infected with bacteria for 4 h, and bacterial invasion was quantified by CFU. (**G**) FLNA siRNA was transfected into 3D4/21 cells, the cells were infected with bacteria for 2 h, and the bacterial adhesion was quantified via CFU.

The entry of bacteria into cells through endocytosis requires the involvement of the actin cytoskeleton (22). To investigate the effect of FLNA on the actin cytoskeleton, we transfected 3D4/21 cells with FLNA siRNA and detected the expression levels of globular actin (G-actin) and filamentous actin (F-actin) in the cells by a G/F-actin Kit. As shown in Fig. 5A through C, FLNA knockdown significantly reduced F-actin production at different time points. Immunofluorescence detection of F-actin showed that FLNA knockdown promoted the depolymerization of F-actin into G-actin and induced the rearrangement of the actin cytoskeleton (Fig. 5D). These results suggest that FLNA knockdown can induce actin cytoskeleton rearrangement. To further investigate the effect of F-actin production on bacterial infection, we treated the cells with two inhibitors that induce actin cytoskeleton rearrangement, Jasplakinolide (which stabilizes actin polymerization) and Latrunculin A (which inhibits actin polymerization), and then F-actin generation levels and bacterial invasion were detected. As shown in Fig. 5E and F, Jasplakinolide treatment increased F-actin production, whereas Latrunculin A decreased F-actin levels. Notably, Jasplakinolide significantly enhanced bacterial invasion, while Latrunculin A markedly suppressed it, suggesting that inhibition of F-actin production could induce actin cytoskeleton rearrangement, thereby suppressing bacterial invasion. To study the effect of FLNA on bacterial adhesion, 3D4/21 cells were transfected with FLNA siRNA, then infected with bacteria, and bacterial adhesion was detected 2 h after infection. As shown in Fig. 5G, FLNA knockdown only inhibited the adhesion of *K. pneumoniae*, but had no significant effect on the adhesion of other bacteria.

### PRRSV promotes bacterial adhesion by upregulating ITGα5 expression

**Fig 6:**
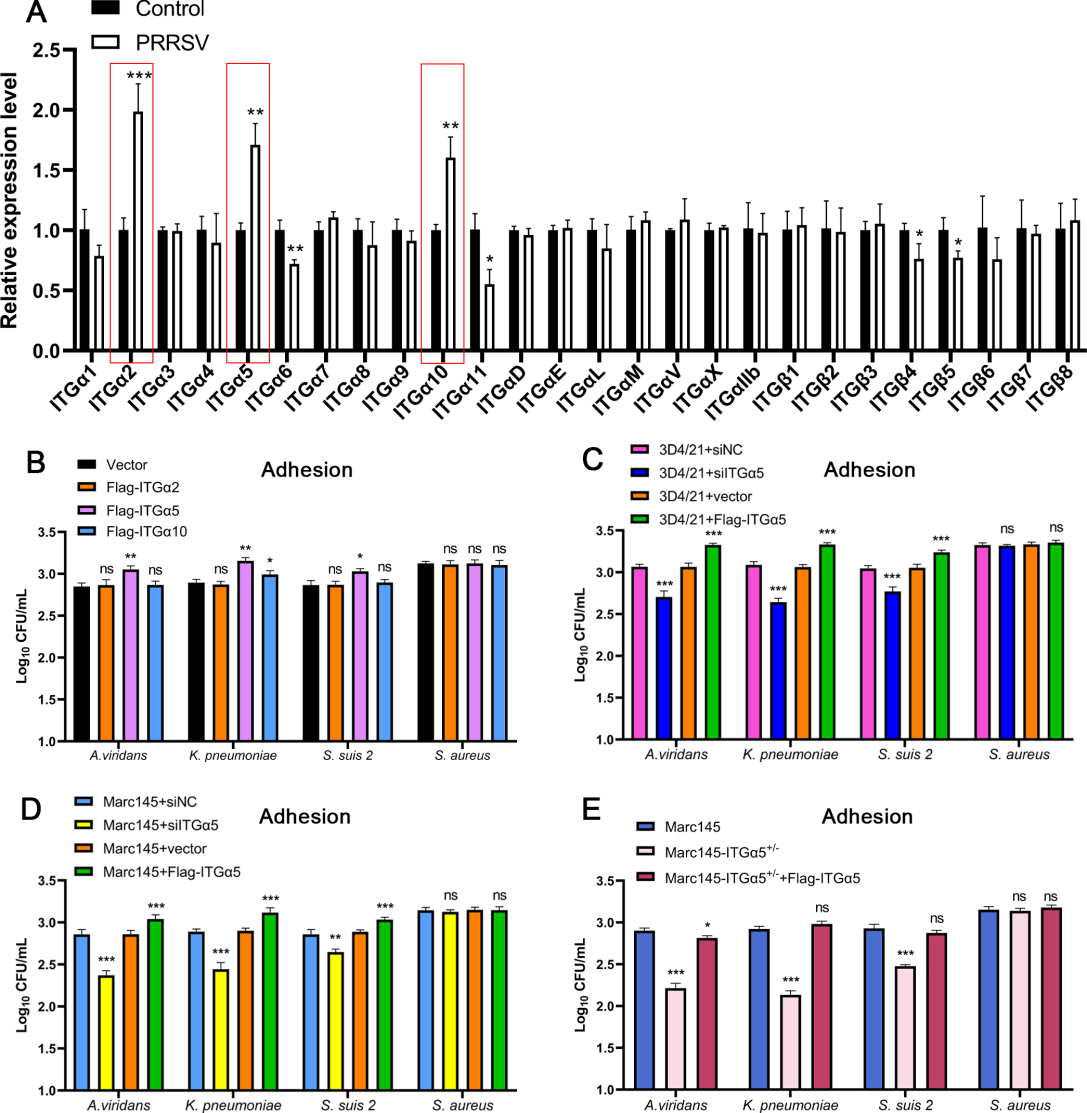
PRRSV promotes bacterial adhesion by upregulating ITGα5 expression. (**A**) 3D4/21 cells were infected with PRRSV, and the expression level of integrin family genes was detected 24 h after infection. (**B**) ITGα2, ITGα5, and ITGα10 overexpression plasmids were transfected into 3D4/21 cells and infected with *A. viridans*, *K. pneumoniae*, *S. suis 2*, or *S. aureus* (MOI = 10) 24 h after transfection, and bacterial adhesion was quantified by CFU. (**C and D**) Transfected 3D4/21 or Marc145 cells with ITGα5 siRNA or overexpressed plasmid for 24 h, infected bacteria, and quantified bacterial adhesion via CFU. (**E**) The ITGα5 overexpression plasmid was transfected into Marc145-ITGα5^+/−^ cells, and then Marc145, Marc145-ITGα5^+/−^, and Marc145-ITGα5^+/−^-Flag-ITGα5 cells were infected with bacteria, and bacterial adhesion was measured using CFU.

Bacterial entry into cells involves two processes: adhesion and invasion, where the bacteria need to first attach to the cell surface and then enter the cell through endocytosis. Bacterial invasion has been explored in the previous sections, but how PRRSV promotes bacterial adhesion remains unclear. It has been reported that integrins are important transmembrane receptors on the cell surface and play an important role in cell adhesion (23). Therefore, we hypothesize that integrins may play an important role in enhancing bacterial adhesion. We infected 3D4/21 cells with PRRSV and examined the expression of 18 α and 8 β subunits of the integrin family. The results showed that the expressions of ITGα2, ITGα5, and ITGα10 were significantly upregulated after PRRSV infection (Fig. 6A). To detect the effect of ITGα2, ITGα5, and ITGα10 on bacterial adhesion, their overexpression plasmids were transfected into 3D4/21 cells, and bacterial adhesion was measured by CFU. The results found that ITGα5 overexpression significantly promoted the adhesion of *A. viridans*, *K. pneumoniae*, and *S. suis 2*, but did not affect the adhesion of *S. aureus*. In addition, the overexpression of ITGα10 also markedly promoted the adhesion of *K. pneumoniae* (Fig. 6B). To further verify the effect of ITGα5 on bacterial adhesion, 3D4/21 and Marc145 cells were transfected with ITGα5 siRNA or an overexpression plasmid. The cells were then infected with bacteria, and the bacterial adhesion was determined by CFU 2 h after infection. As shown in Fig. 6C and D, ITGα5 overexpression significantly increased the adhesion of *A. viridans*, *K. pneumoniae*, and *S. suis 2*, and ITGα5 knockdown suppressed their adhesion. Subsequently, Marc145-ITGα5^+/−^ knockout cells were generated to evaluate bacterial adhesion. The results showed that bacterial adhesion was significantly inhibited in Marc145-ITGα5^+/−^ cells, while reintroduction of ITGα5 expression markedly enhanced bacterial adhesion to the cells (Fig. 6E). These results suggest that PRRSV promotes bacterial adhesion by upregulating ITGα5 expression.

### ITGα5 increases bacterial invasion by promoting F-actin production

**Fig 7:**
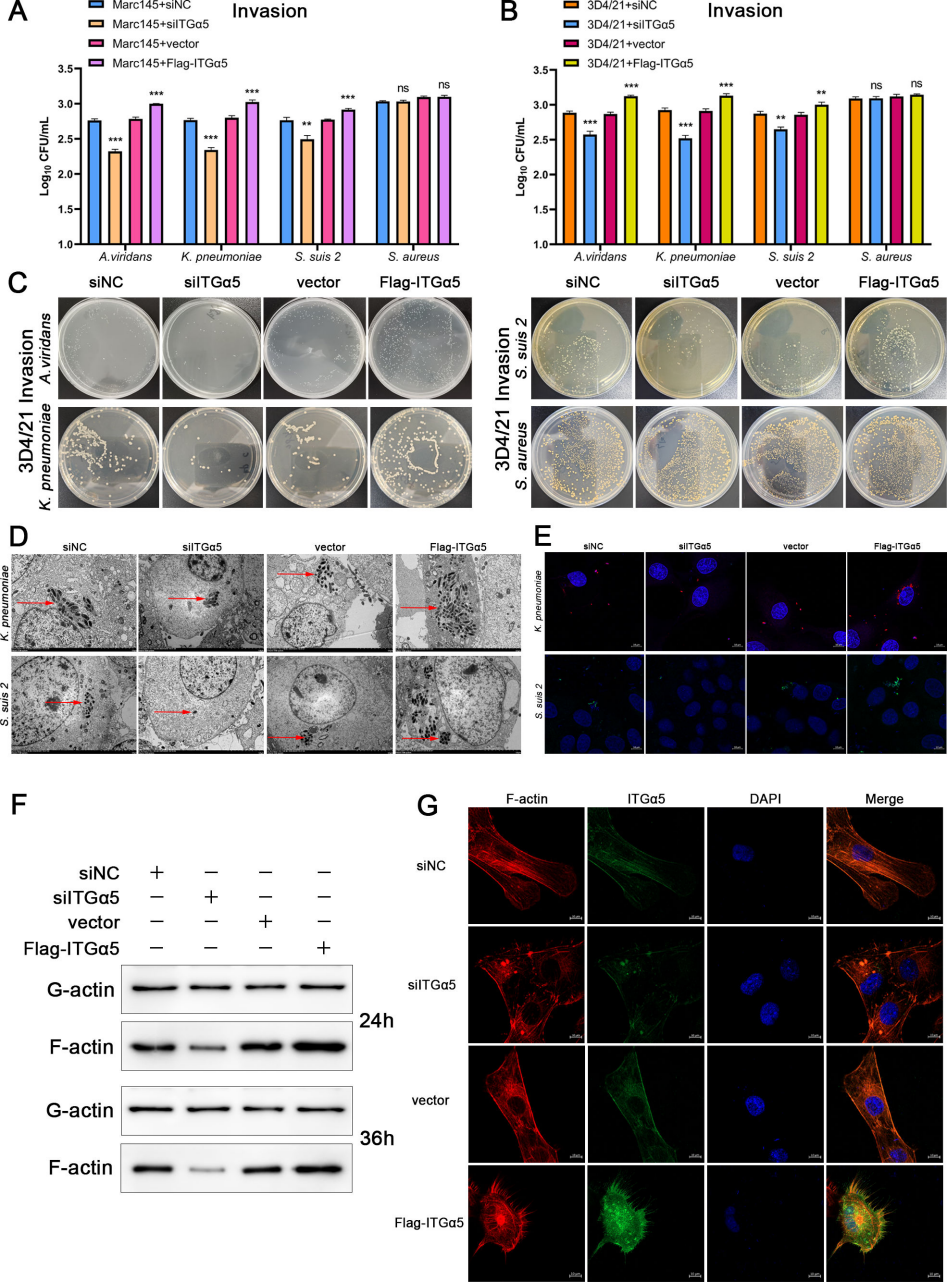
ITGα5 increases bacterial invasion by promoting F-actin production. (**A–C**) Marc145 or 3D4/21 cells were transfected with ITGα5 siRNA or overexpression plasmid for 24 h and infected with *A. viridans*, *K. pneumoniae*, *S. suis 2*, or *S. aureus* (MOI = 10), and bacterial invasion was quantified by CFU. (**D and E**) 3D4/21 cells were transfected with ITGα5 siRNA or overexpression plasmid for 24 h, infected with *K. pneumoniae* or *S. suis 2* for 4 h. Bacterial invasion was observed using transmission electron microscopy and immunofluorescence (red arrowheads indicate bacteria). (**F and G**) ITGα5 siRNA or overexpression plasmid was transfected into 3D4/21 cells, and G/F-actin levels were detected 24 and 36 h after transfection. The cells were collected 24 h after transfection for F-actin immunofluorescence detection.

To further investigate whether ITGα5 influences bacterial invasion, we transfected Marc145 and 3D4/21 cells with ITGα5 siRNA or an overexpression plasmid. The cells were then infected with *A. viridans*, *K. pneumoniae*, *S. suis 2*, or *S. aureus*, and the bacterial invasion was quantified with CFU 4 h after infection. As shown in Fig. 7A through C, the knockdown of ITGα5 significantly reduced the invasion of *A. viridans*, *K. pneumoniae*, and *S. suis 2*, while the overexpression of ITGα5 significantly increased their invasion, but did not affect *S. aureus*. The effect of ITGα5 on bacterial invasion was observed by transmission electron microscopy. As shown in Fig. 7D, ITGα5 knockdown significantly inhibited the invasion of *A. viridans*, *K. pneumoniae*, and *S. suis 2*, while its overexpression promoted their invasion. A similar trend was also obtained when the effect of ITGα5 on bacterial invasion was examined using immunofluorescence (Fig. 7E). To further examine the impact of ITGα5 on G/F-actin levels in 3D4/21 cells, the results revealed that ITGα5 knockdown suppressed F-actin production, while ITGα5 overexpression enhanced F-actin generation (Fig. 7F). Consistent findings were observed via F-actin immunofluorescence (Fig. 7G). Next, we examined the effects of PRRSV, H1N1, and PCV2 infections on ITGα5 expression and found that their infections significantly upregulated ITGα5 expression at distinct time points (Fig. S2). These results indicate that ITGα5 can promote bacterial invasion by inducing actin cytoskeleton rearrangement.

### ITGα5 interacts with FLNA and promotes its expression

**Fig 8:**
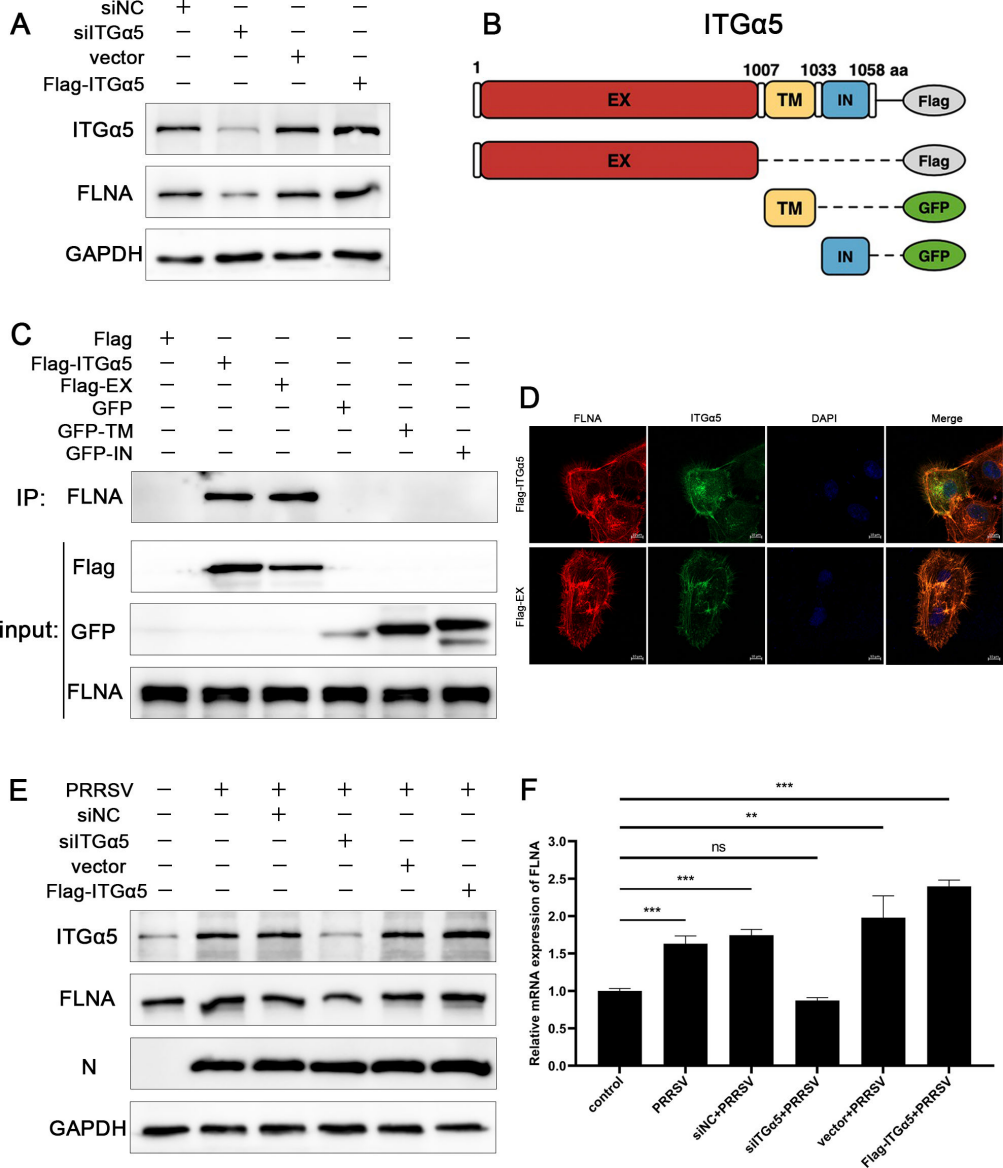
ITGα5 interacts with FLNA and promotes its expression. (**A**) 3D4/21 cells were transfected with ITGα5 siRNA or overexpression plasmid, and FLNA expression was detected 24 h after transfection. (**B and C**) ITGα5 full-length plasmid and single-domain plasmids were transfected into 3D4/21 cells, and the cells were collected for immunoprecipitation analysis. (**D**) 3D4/21 cells were transfected with ITGα5 or ITGα5 extracellular domain plasmid, and cells were collected for colocalization detection. (**E and F**) 3D4/21 cells were transfected with ITGα5 siRNA or overexpression plasmid, and simultaneously inoculated with PRRSV GD-HD (MOI = 1). The expression of FLNA was detected after 36 h infection.

Our above findings indicate that FLNA promotes bacterial invasion by facilitating F-actin generation, and ITGα5 could also enhance bacterial invasion through increasing F-actin production. It has been reported that ITGα5 plays an important role in the regulation of actin cytoskeleton (24), so we hypothesize that ITGα5 may promote bacterial invasion by regulating FLNA expression. To explore whether ITGα5 could regulate FLNA expression, we transfected ITGα5 overexpression plasmid and siRNA into cells to detect the effect of ITGα5 on FLNA expression. The results showed that ITGα5 overexpression significantly promoted FLNA expression, while its knockdown inhibited FLNA expression (Fig. 8A). To determine how ITGα5 promotes FLNA, the ITGα5 full-length plasmid (Flag-ITGα5), extracellular domain (Flag-EX), transmembrane region (GFP-TM), or intracellular domain (GFP-IN) plasmids were subsequently transfected into 3D4/21 cells to identify domains that interact with FLNA. As shown in Fig. 8B and C, both the ITGα5 full-length plasmid and the extracellular domain interacted with FLNA, but the other domains did not, indicating that the extracellular domain is the key domain for interacting with FLNA. Their colocalization was further confirmed via immunofluorescence (Fig. 8D). These results suggest that ITGα5 interacts with FLNA and significantly promotes FLNA expression, thereby promoting bacterial invasion. To further verify the role of ITGα5 in PRRSV promoting FLNA expression, we transfected ITGα5 siRNA or overexpression plasmid into PRRSV-infected 3D4/21 cells and detected FLNA expression 36 h after infection. The results showed that PRRSV infection significantly upregulated FLNA expression, while PRRSV infection combined with ITGα5 siRNA transfection markedly inhibited this FLNA upregulation (Fig. 8E and F), suggesting that ITGα5 is a key mediator in PRRSV-induced FLNA expression enhancement.

### Knockdown of FLNA and ITGα5 inhibited bacterial infection in the lungs of mice

**Fig 9:**
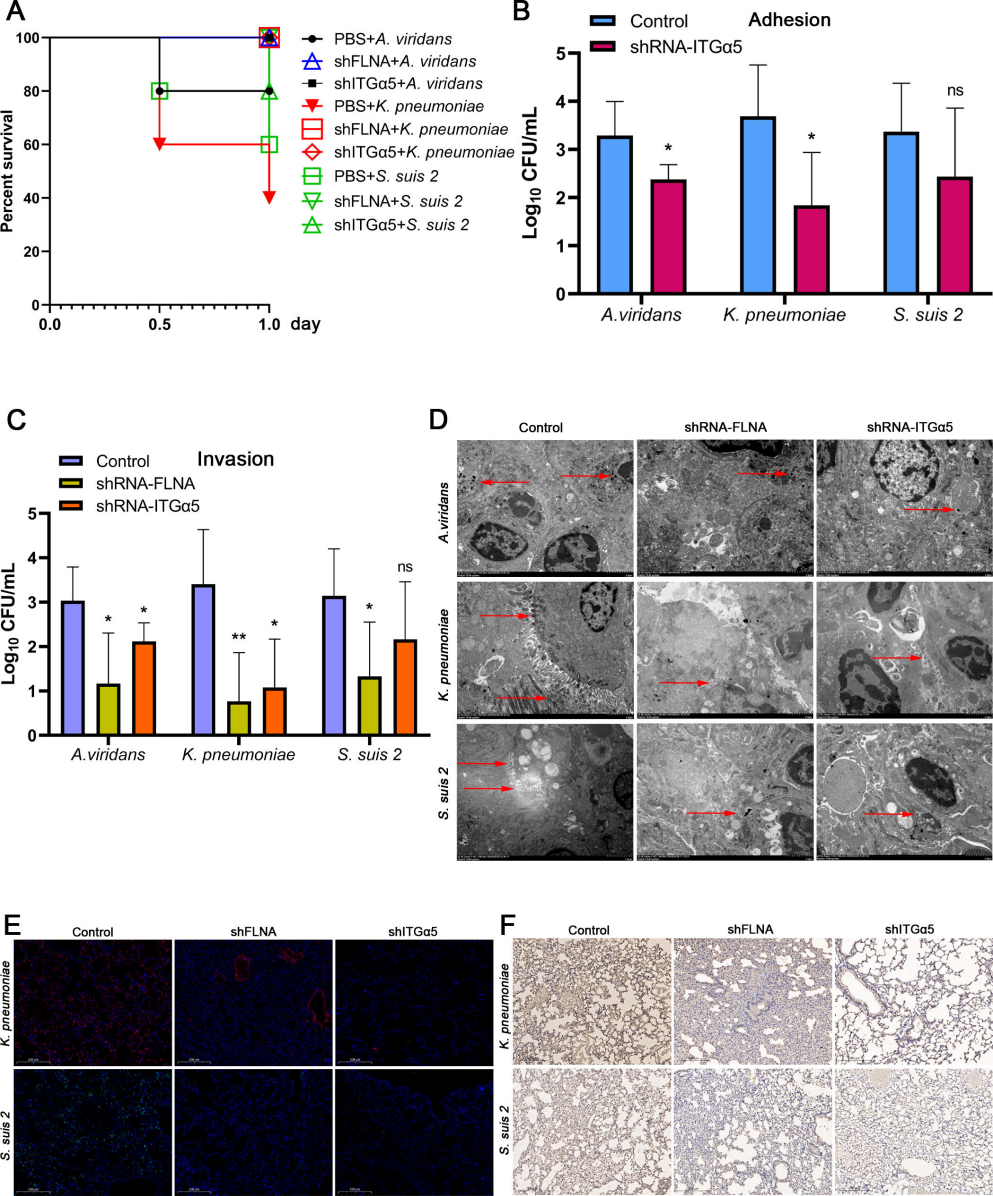
Knockdown of FLNA and ITGα5 inhibited bacterial infection in the lungs of mice. (**A**) Survival curves of the mice after bacterial infection. (**B and C**) After the mice died or were sacrificed, their lungs were homogenized, and then bacterial adhesion or invasion was quantified by CFU. (**D**) Lung sections were prepared, and the bacteria were observed by transmission electron microscopy (red arrowheads indicate bacteria). (**E and F**) Immunofluorescence and immunohistochemistry assays were performed with anti-*K*. *pneumoniae* and anti-*S*. *suis 2* antibodies.

To further verify the effects of FLNA and ITGα5 on bacterial infection *in vivo*, we constructed shRNA-mediated knockdown mouse models by the pLKO.1 lentiviral vector, then infected these mice with *A. viridans*, *K. pneumoniae*, or *S. suis 2*. The PBS + bacteria groups of mice began to die on Day 0.5 after bacterial infection (Fig. 9A and Table 2), and all mice were sacrificed on Day 1. Detection of FLNA and ITGα5 expression in the lungs of mice showed that shRNA lentivirus infection significantly reduced their expression levels (Fig. S3). Quantification of bacterial adhesion using CFU showed that knockdown of ITGα5 significantly reduced the adhesion of *A. viridans*, *K. pneumoniae*, and *S. suis 2* in murine lungs (Fig. 9B). Quantification of bacterial invasion by CFU revealed that knockdown of FLNA or ITGα5 markedly decreased the loads of these three bacterial species (Fig. 9C), and a similar trend was observed by transmission electron microscopy (Fig. 9D). Subsequent immunofluorescence and immunohistochemistry analyses of *K. pneumoniae* and *S. suis 2* infections in the lungs of mice demonstrated that knockdown of either FLNA or ITGα5 significantly reduced pulmonary bacterial burden (Fig. 9E and F). These results suggest that FLNA and ITGα5 play critical roles in bacterial infection in mice.

**TABLE 2:** Survival statistics of mice before sacrifice

Group	Survival	Death
PBS + *A. viridans*	4	1
shITGα5 + *A. viridans*	5	0
shFLNA + *A. viridans*	5	0
PBS + *K. pneumoniae*	2	3
shITGα5 + *K. pneumoniae*	5	0
shFLNA + *K. pneumoniae*	5	0
PBS + *S. suis 2*	3	2
shITGα5 + *S. suis 2*	4	1
shFLNA + *S. suis 2*	5	0

### PRRSV inhibits the transcription factor HOXD8 to promote ITGα5 expression

**Fig 10:**
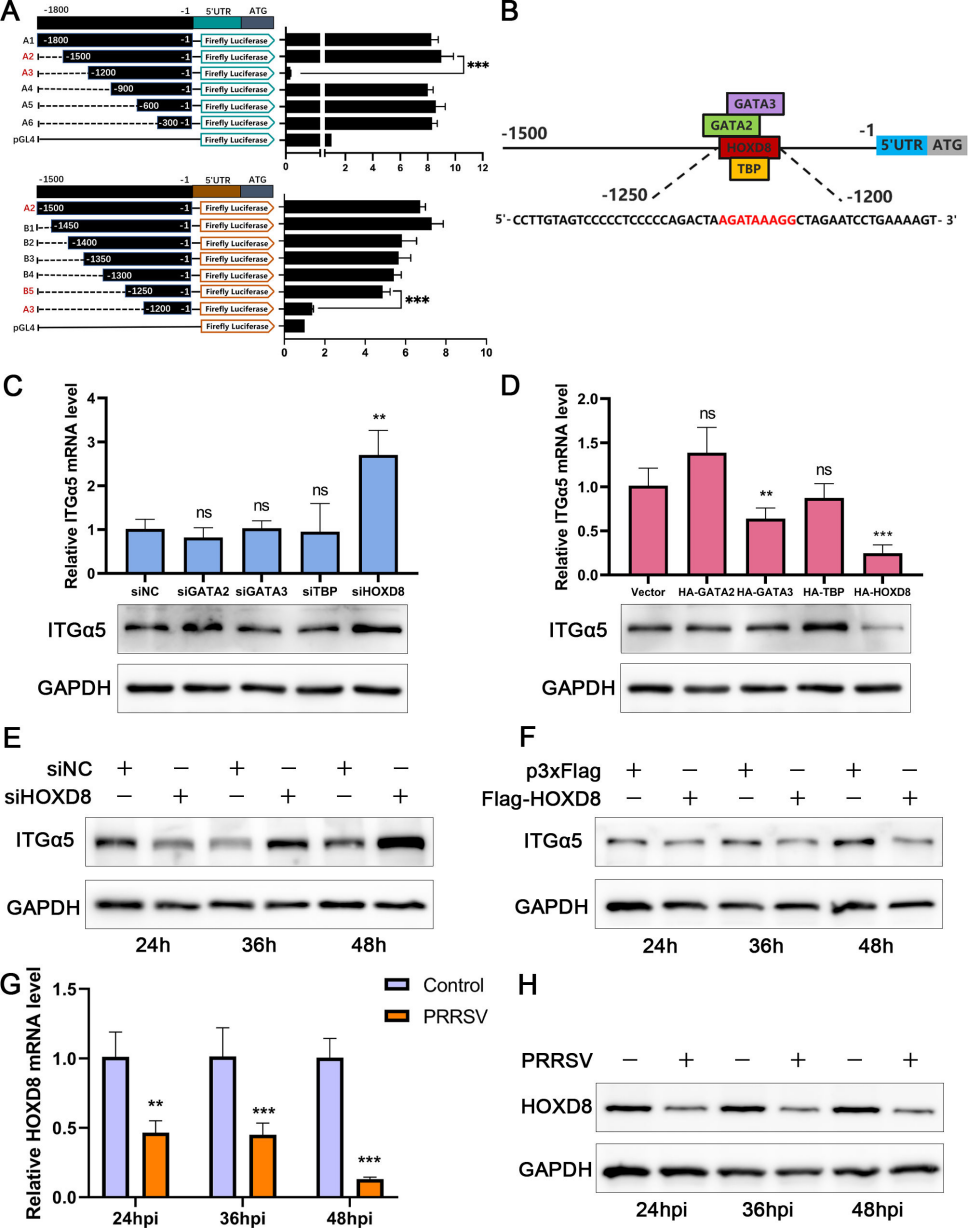
PRRSV inhibits the transcription factor HOXD8 to promote ITGα5 expression. (**A**) The ITGα5 promoter region plasmids were co-transfected with pRL-TK into 3D4/21 cells, and the activity was analyzed by a dual-luciferase reporter kit. (**B**) PROMO was used to predict transcription factors that could bind to the target sequence. (**C and D**) Marc145 cells were transfected with siRNA or overexpression plasmids of the predicted transcription factors for 24 h, and ITGα5 expression was detected. (**E and F**) Marc145 cells were transfected with HOXD8 siRNA or overexpression plasmid, and the cells were collected for Western blot analysis. (**G and H**) Marc145 cells were infected with PRRSV GD-HD (MOI = 1), and the cells were harvested to measure the expression of HOXD8.

To explore how PRRSV upregulates the expression of ITGα5, we studied the transcriptional regulatory mechanism of ITGα5 and identified the essential cis-regulatory elements of the ITGα5 promoter. The −0.3, −0.6, −0.9, −1.2, −1.5, and −1.8 kb regions were segmented to construct the PGL4.10 vector, which was subsequently transfected into 3D4/21 cells to assess promoter activity (25). As shown in Fig. 10A, there was a significant decrease in promoter activity from −1.5 kb to −1.2 kb, indicating the presence of key active sites in this region. We further truncated the region and identified the ITGα5 promoter in the −1.25 kb to −1.2 kb region. The sequence was submitted to the bioinformatic tool PROMO to predict transcription factor binding sites (Fig. 10B). Knockdown and overexpression of the predicted transcription factors were performed, and the expression level of ITGα5 was detected. The results showed that HOXD8 knockdown significantly promoted the expression of ITGα5, while overexpression markedly inhibited its expression, indicating that HOXD8 is a negative transcriptional regulator of ITGα5 (Fig. 10C and D). Consistent results were also obtained for HOXD8 knockdown and overexpression at different time points (Fig. 10E and F). Next, we examined HOXD8 expression level after PRRSV infection, and the results showed that PRRSV infection significantly reduced HOXD8 expression at different time points (Fig. 10G and H). These results indicated that HOXD8 is a negative transcriptional regulator of ITGα5, and PRRSV promoted ITGα5 expression by inhibiting the expression of HOXD8.

### PRRSV Nsp10 interacts with HOXD8 and decreases its expression

**Fig 11:**
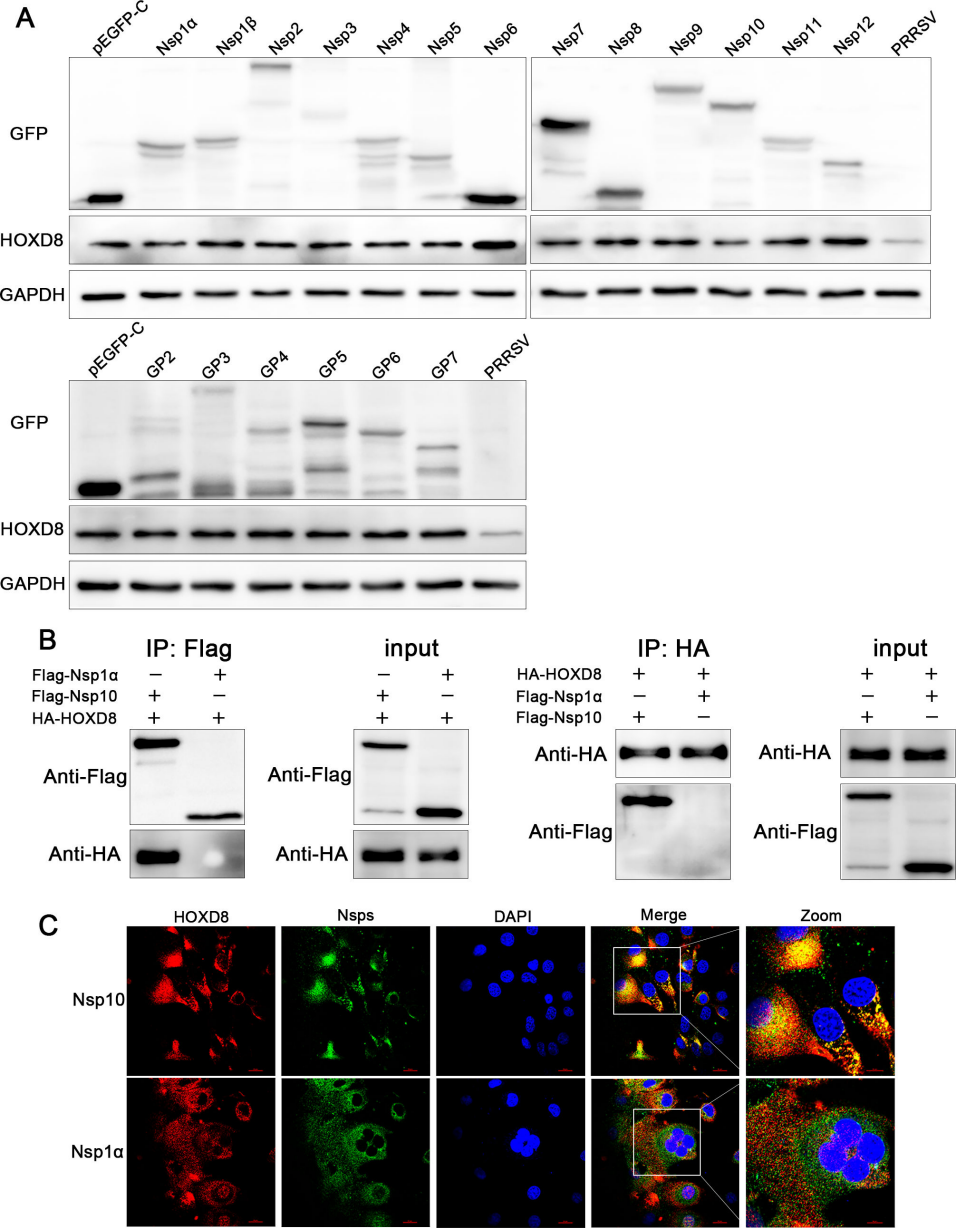
PRRSV Nsp10 interacts with HOXD8 and decreases its expression. (**A**) PRRSV nonstructural and structural protein plasmids were transfected into 3D4/21 cells, and the cells were collected 24 h later to detect the expression of viral proteins and HOXD8. (**B**) HOXD8 was co-transfected into HEK-293T cells with PRRSV Nsp10 or Nsp1α, and the cells were harvested 24 h later for coimmunoprecipitation analysis. (**C**) Marc145 cells were infected with PRRSV GD-HD (MOI = 1), and the colocalization of HOXD8 with Nsp10 or Nsp1α was detected by immunofluorescence at 24 h.

To study the major viral fragment that downregulated HOXD8 expression, nonstructural and structural proteins of PRRSV were transfected into 3D4/21 cells, and the expression level of HOXD8 was detected after 24 h. The results showed that Nsp10 was the most significant fragment that inhibited HOXD8 expression (Fig. 11A). We subsequently co-transfected HEK-293T cells with HOXD8 and PRRSV Nsp10 or Nsp1α to perform coimmunoprecipitation assays 24 h after transfection. As shown in Fig. 11B, Nsp10 interacts with HOXD8, whereas Nsp1α does not. Next, we infected Marc145 cells with PRRSV and detected the colocalization of Nsp10 and HOXD8 by immunofluorescence 24 h after infection. The results showed that Nsp10 was colocalized with HOXD8 (Fig. 11C).

## DISCUSSION

Bacterial adhesion and colonization are prerequisites for the establishment of infection. Secondary bacterial infection caused the majority of associated deaths during the SARS-CoV-2 pandemic and influenza outbreaks (26–28). As a precursor to infection, viruses may damage bronchial and alveolar epithelial cells, paving the way for bacterial infection (29, 30). Studies have reported that neuraminidase (NA) of influenza A virus (IAV) activates the TGF-β signaling pathway and promotes the expression of cellular adhesins, leading to an increase in bacterial colonization and promoting secondary bacterial infection (31). In this study, we found that PRRSV, H1N1, and PCV2 infections could significantly aggravate multiple bacterial infections, and infections of PRRSV, H1N1, and PCV2 all significantly increased the expressions of FLNA and ITGα5. We found that PRRSV can upregulate the expression of ITGα5 by inhibiting the expression of HOXD8 (Fig. 10), and H1N1 and PCV2 can also suppress the expression of HOXD8 (data not shown). These results suggest that this might be a conserved mechanism by which respiratory viruses exacerbate bacterial infections (32, 33). *In vivo* experiments showed that FLNA and ITGα5 knockdown significantly reduced pulmonary bacterial burden in the lungs of mice. These results suggest that FLNA and ITGα5 are critical factors for bacterial infection and that the regulation of actin cytoskeleton and cell membrane proteins may be a mediator of virus-bacteria interactions.

Bacterial superinfection following virus infection is a common complication (34), and *K. pneumoniae* is one of the most harmful pathogens, often leading to severe pneumonia, and has become a serious public health threat worldwide with the spread of drug-resistant strains (35). We observed that the knockdown of ITGα5 almost completely eliminated *K. pneumoniae* infection, whereas cell susceptibility to *K. pneumoniae* was restored after the ectopic expression of ITGα5. These findings indicate that ITGα5 may be an adhesion receptor for *K. pneumoniae* infection and a bridge for its infection.

We found that ITGα5 can induce actin cytoskeleton rearrangement by upregulating FLNA expression, thereby facilitating bacterial invasion. The amount of F-actin and G-actin in the cell membrane is dynamically stable in the normal physiological state, which is an essential condition for maintaining cell endocytosis and movement (36, 37). Any pathological factor that disrupts this homeostasis may aggravate pathogenic infection (38). The endocytosis of cells can be used by the virions of Hendra virus and Newcastle disease virus to increase their own entry and infection efficiency (39), while the T3SS translocation of SipC and the effector SipA are crucial for Salmonella infection by disrupting the host cytoskeleton (22). These studies indicate that the regulation of the actin cytoskeleton by viruses is an important factor leading to increased pathogen infection.

**Fig 12:**
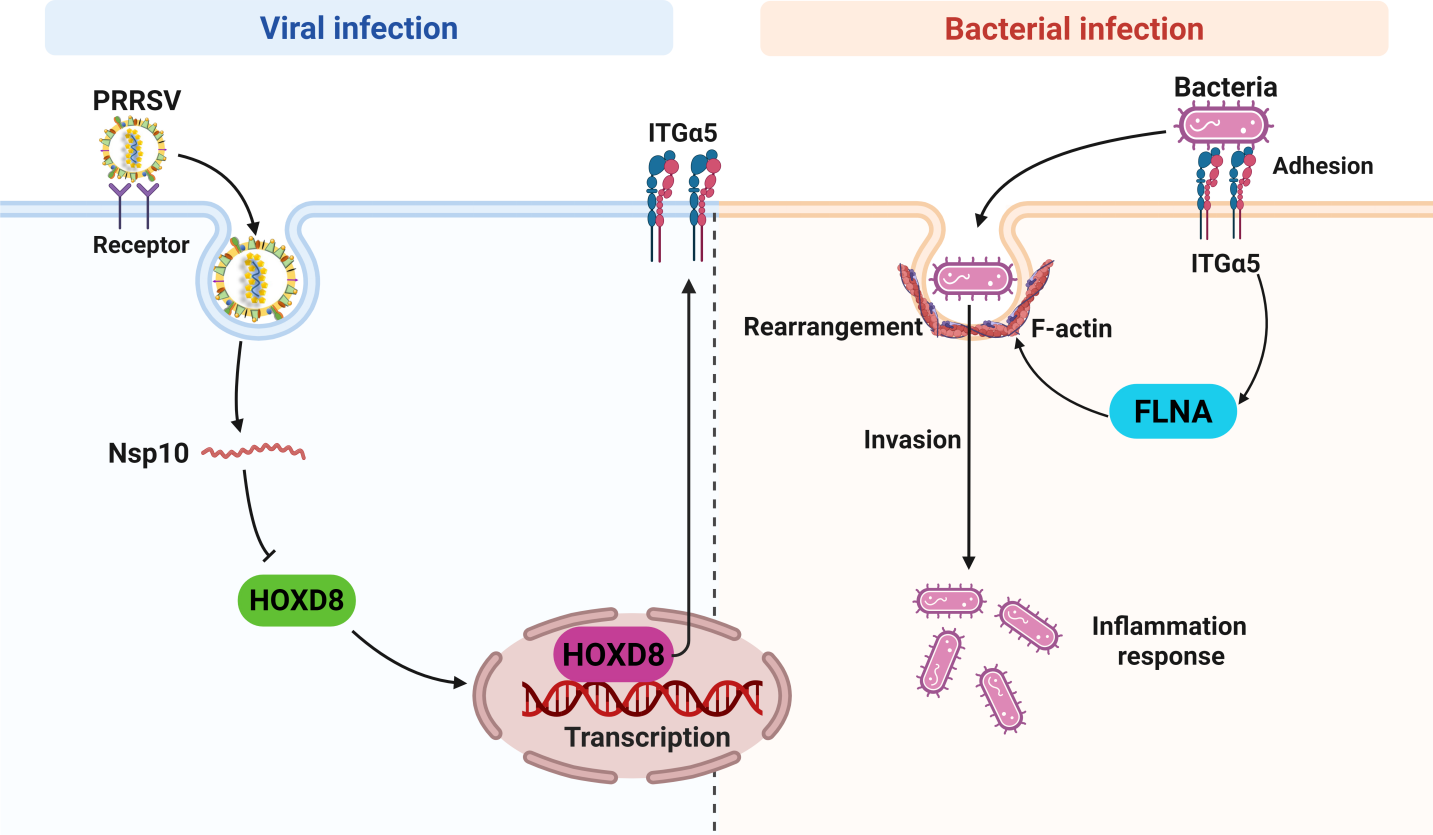
Mechanism model of PRRSV promoting secondary bacterial infection. PRRSV Nsp10 promoted ITGα5 expression by suppressing the expression of the transcription factor HOXD8, thus facilitating bacterial adhesion. The upregulation of ITGα5 increased FLNA expression and induced actin cytoskeleton rearrangement, thereby promoting bacterial infection. The schematic was drawn by the Biorender website.

In summary, we found that PRRSV induced actin cytoskeleton rearrangement by upregulating FLNA expression, thereby aggravating bacterial invasion. PRRSV increased bacterial adhesion by promoting the ITGα5 expression, and the upregulation of ITGα5 could induce FLNA-mediated actin cytoskeleton rearrangement. The promotion of ITGα5 expression by PRRSV is dependent on the repression of the transcription factor HOXD8 (Fig. 12). Furthermore, we found that H1N1 and PCV2 infection also significantly promoted the expression of FLNA and ITGα5 and increased the infection of multiple bacteria. Lentiviral shRNA-mediated knockdown of FLNA or ITGα5 significantly reduced bacterial infection in the lungs of mice and protected mice from death. Our research indicates that the regulation of actin cytoskeleton and cell membrane proteins during viral infection is critical for secondary bacterial infection, which provides a new perspective for the control of secondary bacterial infection in the clinic.

## MATERIALS AND METHODS

### Virus, bacteria, and cells

The PRRSV GD-HD strain (GenBank: KP793736) and porcine circovirus type 2d (PCV2) SX-XY18 strain (GenBank: PP668228) used in this study were isolated and preserved in our laboratory (Lanzhou Veterinary Research Institute, Lanzhou, China) (40). The influenza A virus (H1N1) SN13 strain (GenBank: MN418766) (41) was gifted by Professor Honglei Sun from China Agricultural University. The *A. viridans*, *K. pneumoniae*, *S. suis 2*, and *S. aureus* strains were isolated from clinical samples and stored in our laboratory. PAMs were isolated from 5-week-old PRRSV antigen and antibody-negative piglets and cultured in RPMI 1640 medium supplemented with 10% fetal bovine serum (FBS). Marc145, 3D4/21, HEK-293T, MDCK, and PK-15 cells were cultured in Dulbecco's Modified Eagle Medium (DMEM) containing 10% FBS at 37°C with 5% CO_2_.

### Antibodies and reagents

Anti-PRRSV N, anti-*S*. *suis 2* monoclonal antibodies and Nsp1α, Nsp10 polyclonal antibodies were prepared and stored in our laboratory. Anti-GAPDH, anti-HA, anti-Flag, and anti-GFP monoclonal antibodies were obtained from TransGen Biotech. Anti-HOXD8 (A16877) polyclonal antibodies were obtained from ABclonal Technology. Anti-ITGα5 (98204), anti-p-AKT (4060), and anti-FLNA (44873) monoclonal antibodies were obtained from Cell Signaling Technology. Anti-ITGα5 (Ab150361) monoclonal antibodies and anti-*K*. *pneumoniae* antibodies (Ab20947) were obtained from Abcam. Anti-AKT (51077-1), anti-mouse IgG (labeled with Alexa Fluor 488), and anti-rabbit IgG (labeled with Alexa Fluor 594) antibodies were obtained from Proteintech. TRITC phalloidin was purchased from Solarbio Technology, a G/F-actin Kit (BK037) was obtained from Cytoskeleton, Inc., and anti-Flag and anti-HA magnetic beads and inhibitors were purchased from MedChemExpress. The anti-FLNA (MA5-11705) monoclonal antibody, Lipofectamine RNAiMAX transfection reagent, and Lipofectamine 3000 transfection reagent were obtained from Thermo Fisher Scientific.

### Bacterial adhesion assay

Cells were seeded in 12-well plates, infected with PRRSV (MOI = 1) for 36 h, or transfected for 24 h. Then, the medium was replaced with serum-free DMEM, and the cells were infected with bacteria (MOI = 10) for 2 h at 37°C with 5% CO_2_. Then washed with PBS to remove unbound bacteria, the cells were digested with trypsin-ethylenediaminetetraacetic acid solution and lysed with 0.25% Triton X-100. After dilution with 1 mL of PBS, bacteria were quantified by CFU.

After infection with bacteria for 24 h, the mice or piglets were sacrificed, the mouse lungs were homogenized in 3 mL of PBS, and piglet lung samples were collected from three sites (1 g at each site) and homogenized in 3 mL of PBS. The homogenate was collected and centrifuged, then the cells were resuspended and lysed with 0.25% Triton X-100. After dilution with PBS, bacteria were quantified by CFU (42).

### Bacterial invasion assay

Cells were seeded in 12-well plates, infected with PRRSV (MOI = 1) for 36 h, or transfected for 24 h, and then infected with bacteria (MOI = 10) for 4 h at 37°C with 5% CO_2_. Then washed with PBS, the cells were incubated with DMEM containing 100 μg of gentamicin and 5 μg of penicillin/mL for 2 h to kill surface-adherent bacteria. After being washed with PBS three times, the intracellular bacteria were quantified by CFU as described in the adhesion assay.

After being infected with bacteria for 24 h, the mice were sacrificed, and the lungs were homogenized in 3 mL of PBS. The homogenate was collected and centrifuged. PBS containing 100 μg of gentamicin and 5 μg of penicillin/mL was used to resuspend the cells, then incubated at 37°C with 5% CO_2_ for 2 h and washed with PBS before quantifying intracellular bacteria (42).

### Quantitative PCR

Total RNA was extracted from cells or tissues via TRIzol. cDNA was obtained by PrimeScript RT Kit, and reverse transcription quantitative real-time polymerase chain reaction (RT-qPCR) analysis was subsequently performed with ChamQ SYBR qPCR Master Mix. The relative expression levels were calculated by the 2^−ΔΔCT^ method. Primers are shown in Table 3.

**TABLE 3:** List of primers used in the RT-qPCR analysis

Primer	Sequence (5′→3′)	Species
gmGAPDH-F	TGGAAAAACCTGCCAAGTACG	Green monkey
gmGAPDH-R	ATGAGGTCCACCACCCTGTT	
pGAPDH-F	TGACAACTCCCTCAAGATCG	Pig
pGAPDH-R	AAGCAGGGATGATGTTCTGG	
dGAPDH-F	TGCCAAATATGACGACATCAA	Dog
dGAPDH-R	GACCACCTGGTCCTCAGTGTA	
gmITGα5-F	GTTTTAGGTGGACCAGGAAGC	Green monkey
gmITGα5-R	GATCGGATGTCTGAGCCATTA	
pITGα5-F	CTGTGCTCCCCTCTACAGTTG	Pig
pITGα5-R	AGAATCCGGGTGAAGTTGTCT	
dITGα5-F	GGGAGGACTGCAGAGAGATG	Dog
dITGα5-R	CGCAGATGTTGTCTTCTCCA	
gmFLNA-F	CATGTCACTGCCTATGGACCT	Green monkey
gmFLNA-R	TGGGATGTGCTGTTCATTGTA	
pFLNA-F	GTCCCTGTGCATGATGTGAC	Pig
pFLNA-R	GGGCACATAGTTGACGGTCT	
dFLNA-F	AAGGTATACGGGCCTGGAGT	Dog
dFLNA-R	CTCCACGGGGCGTATACTTC	
gmHOXD8-F	AGCAGCTCCTGGTAGACGAA	Green monkey
gmHOXD8-R	TTAATTTGTCGGGCCTTCTG	
PRRSV-ORF7-F	AATGGCCAGCCAGTCAATCA	PRRSV
PRRSV-ORF7-R	TCATGCTGAGGGTGATGCTG	
pITGα1-F	TATGCCCTGAATCAGACAAGG	Pig
pITGα1-R	TGCATGAATTGTGCTTCAGAG	
pITGα2-F	GTGCCTTTGGACAGGTTGTT	Pig
pITGα2-R	TCATGGTCTTCTGCAAGCAC	
pITGα3-F	ACATCTACCACGGCAGTTCC	Pig
pITGα3-R	CACTCAGGGAGTAGCCGAAG	
pITGα4-F	AACATGAGCCTGGATGTGAAC	Pig
pITGα4-R	GCTGAAGAATTGGCTGAAGTG	
pITGα6-F	GGCCTTATGAAGTTGGTGGA	Pig
pITGα6-R	CCACCACTGCCACATCATAG	
pITGα7-F	AGCAGAGGAGCTGAGCTTTG	Pig
pITGα7-R	GTAGGGAGCACCCACTACCA	
pITGα8-F	GGCAGATACCCTTTGACAACA	Pig
pITGα8-R	CTGTTGCTCCAAACCATTGAT	
pITGα9-F	ATCACGTCTCCAACCTCCTTT	Pig
pITGα9-R	ATGACACTCCAGGTCATCCAG	
pITGα10-F	GTGAGAGCTTCCTGGAGGTG	Pig
pITGα10-R	CAAGCTTCCAAAGGCAAAAG	
pITGα11-F	ATGGCGTGACTGATGTCCTAC	Pig
pITGα11-R	CTGCTTTGGTGTCTTCAGGAG	
pITGαD-F	AGATCCGTGTATTCCCAGCTT	Pig
pITGαD-R	ACGGGGTTATGGACCTCATAC	
pITGαE-F	CGCAGAGCTCTCCTTAAGTCA	Pig
pITGαE-R	CCCAACACTGCTTTGAATGAT	
pITGαL-F	GTCAGCCAGACAATGGACAAT	Pig
pITGαL-R	TGGTGGAAGAGGTAACACAGG	
pITGαM-F	AGAAGGAGACACCCAGAGCA	Pig
pITGαM-R	GTAGGACAATGGGCGTCACT	
pITGαV-F	GCAGAAAGGAGCAATTCGAC	Pig
pITGαV-R	GGGTTGCAAGCCTGTTACAT	
pITGαX-F	GCTCCTTCGAGTTGGAGATG	Pig
pITGαX-R	ACCAGGCTCTGTACCCCTTT	
pITGαIIb-F	TGCTCAGCTTCAATGTGTCC	Pig
pITGαIIb-R	CACTTGGTCTTCCCCACAGT	
pITGβ1-F	GGATTTGGCTCTTTTGTGGA	Pig
pITGβ1-R	CCACCTTCTGGAGAATCCAA	
pITGβ2-F	CTGAACTTCTCTGGGCAAGG	Pig
pITGβ2-R	TGTCACTTTCTGTGGGGACA	
pITGβ3-F	AAGAAGGGGTGTGTGGAGTG	Pig
pITGβ3-R	TGGGACACTCTGGCTCTTCT	
pITGβ4-F	ACGTGTGTTCCTGTGAGCTG	Pig
pITGβ4-R	GGGACACTGGAAGTTGTCGT	
pITGβ5-F	GTCCTCAAGGAGCCAGAGTG	Pig
pITGβ5-R	TCCACGTTGTGTGTGGAGAT	
pITGβ6-F	TGCGACCATCAGTGAAGAAG	Pig
pITGβ6-R	TAGCCTTTGACCGTTCTGCT	
pITGβ7-F	TCGGCTCTCAGTGGAAATCTA	Pig
pITGβ7-R	GTTCTGCTTCCATTTGAGCTG	
pITGβ8-F	TGCAGCTGTCAATGTGATGA	Pig
pITGβ8-R	GCACACAGATTTCCATGGTG	

### Western blot and coimmunoprecipitation

The cells were collected and lysed, and the protein content of the samples was quantified by a BCA Kit. Then, the proteins were denatured in boiling water for 10 min, separated via 12% SDS-PAGE, transferred to polyvinylidene fluoride (PVDF) membrane, blocked in 5% skim milk at room temperature for 1 h, incubated with primary antibodies at 4°C overnight, incubated with secondary antibodies at room temperature for 1 h, and incubated with enhanced chemiluminescence (ECL) solution to detect the protein bands.

The magnetic beads were pretreated with PBST, and the whole-cell lysates were added to the magnetic beads and incubated at room temperature for 3 h. The beads were rinsed with PBST five times, then prepared samples for Western blot analysis. Occasionally, the edge of the blot can be seen as a non-uniform white background in the image because the bands were too close to the edge of the membrane.

### Immunofluorescence

Cells were fixed with 4% paraformaldehyde for 30 min, permeabilized with 0.1% Triton X-100 at room temperature for 7 min, blocked with 3% bovine serum albumin (BSA) for 1 h, and incubated with primary antibodies at room temperature for 2 h. After being washed with PBS, the cells were incubated with fluorescent secondary antibodies for 1 h, rinsed with PBS, and then stained with 4′,6-diamidino-2′-phenylindole (DAPI) for 6 min. An antifluorescence quencher was added, and immunofluorescence was observed with a Zeiss fluorescence microscope.

### Plasmid construction

The coding sequences of the target genes were obtained either by downloading sequences from the NCBI database followed by commercial gene synthesis, or by PCR amplification. These sequences were subsequently cloned into the appropriate expression vectors: the lenti-cmv-mcs-Flag vector was used to generate Flag-ITGα2, Flag-ITGα5, Flag-EX, and Flag-ITGα10; the pEGFP-C vector was used for constructing GFP-TM, GFP-IN, GFP-Nsp1α-Nsp12, and GFP-GP2-GP7; the pcaggs-Flag vector was employed to create Flag-HOXD8, Flag-Nsp1α, and Flag-Nsp10; and the pcsggs-HA vector was used to generate HA-HOXD8. All final plasmid constructs were verified by commercial Sanger sequencing.

### Sample preparation and RNA-seq analysis

PAMs were seeded in 60 mm culture dishes at a density of 1 × 10⁶ cells per dish, and the cells were infected with PRRSV GD-HD (MOI = 1), with uninfected cells serving as controls. Samples were collected with TRIzol reagent at different time points of infection and then sent to the company to extract total RNA for RNA-seq analysis. Transcriptome sequencing was performed by Tsingke Biotechnology Co., Ltd. (Beijing, China), and bioinformatic analysis was conducted using the OmicStudio tools.

### G/F-actin detection

The cells were collected and added to 100 µL LAS2 buffer and incubated at 37°C for 10 min. The samples were subsequently centrifuged at 350 × *g* at room temperature for 5 min, after which the supernatant was collected and centrifuged at 100,000 × *g*, 37°C for 1 h. The G-actin remained in the supernatant, while the pellet contained F-actin. The supernatant was transferred to a fresh tube, mixed with SDS sample buffer, and denatured in boiling water for 10 min. The pellet was resuspended in 100 µL of F-actin depolymerization buffer and incubated on ice for 1 h. SDS sample buffer was added, followed by denaturation in boiling water for 10 min. Then, the expression of G/F-actin was analyzed via Western blotting.

### Lentiviral shRNA-mediated knockdown mouse models

shRNA was constructed in the pLKO.1 lentiviral vector, and the knockdown sequences for FLNA and ITGα5 were inserted into the vector. The shRNA plasmid was co-transfected into HEK-293T cells with the helper plasmids L-M and L-S, and the supernatant was collected 48 h later for infection of cells or mice. A total of 300 µL supernatant was used to infect the mice by intramuscular injection into the back, and the mice were infected again 48 h later. FLNA or ITGα5 knockdown mouse models were generated 14 days after infection (43, 44).

### Bacterial infection

Fifteen 4-week-old BALB/c mice were divided into control, shFLNA, and shITGα5 groups, with five mice in each group. Mice were infected with pLKO.1 empty vector lentivirus or shRNA lentivirus, and 2 weeks later, the mice were injected intraperitoneally with *K. pneumoniae* (1 × 10^6^ CFU) (45). The infection experiments involving *A. viridans* and *S. suis 2* were conducted using the same grouping and experimental methods.

### Piglet infection experiment

Twenty-five 4-week-old piglets negative for PRRSV antigen and antibodies were purchased from a pig farm. None of the pigs were vaccinated against PRRSV. The piglets were randomly divided into five groups: PRRSV + *K. pneumoniae*, *K. pneumoniae*, PRRSV + *S. suis 2*, *S. suis 2*, and PRRSV, with five pigs in each group, housed individually. When piglets are infected with PRRSV GD-HD, their body temperature increases rapidly and is maintained for a period of time after PRRSV infection. When the body temperature returned to normal, the piglets were infected with 1 mL (5 × 10^8^ CFU) of *K. pneumoniae* or *S. suis 2* (46). The piglets were sacrificed 24 h after bacterial infection, and necropsy and gross pathological examination of the lungs were immediately performed with photographic documentation. Lung tissues were then collected for lung lavages to isolate PAMs for western blotting (WB) analysis, and the lungs were taken for bacterial quantification by CFU.

### Statistical analysis

The experiments were repeated three times and statistically analyzed via GraphPad Prism 8 software. The results are expressed as the means ± standard deviations. The significance of differences was analyzed via Student’s *t*-test or one-way analysis of variance, and *P* values of <0.05 (*), <0.01 (**), and <0.001 (***) were considered statistically significant at different levels. “ns” indicates no significant difference.

## Data Availability

Transcriptomics data have been deposited in the Sequence Read Archive (SRA) of the NCBI database, under BioProject accession number PRJNA1309714.

## SUPPLEMENTAL MATERIAL

Supplemental Material mbio.01945-25-s0001.docx Fig. S1 to S3.

ASM does not own the copyrights to Supplemental Material that may be linked to, or accessed through, an article. The authors have granted ASM a non-exclusive, world-wide license to publish the Supplemental Material files. Please contact the corresponding author directly for reuse.
