# Supplementary material for: PRRSV promotes bacterial infection by remodeling actin cytoskeleton and cell membrane proteins
Source: mBio. 2025 Sep 12;16(10):e01945-25. doi: 10.1128/mbio.01945-25 (PMC12506063; doi:10.1128/mbio.01945-25)
Supplement: Supplemental Material — Fig. S1 to S3. [file mbio.01945-25-s0001.docx]

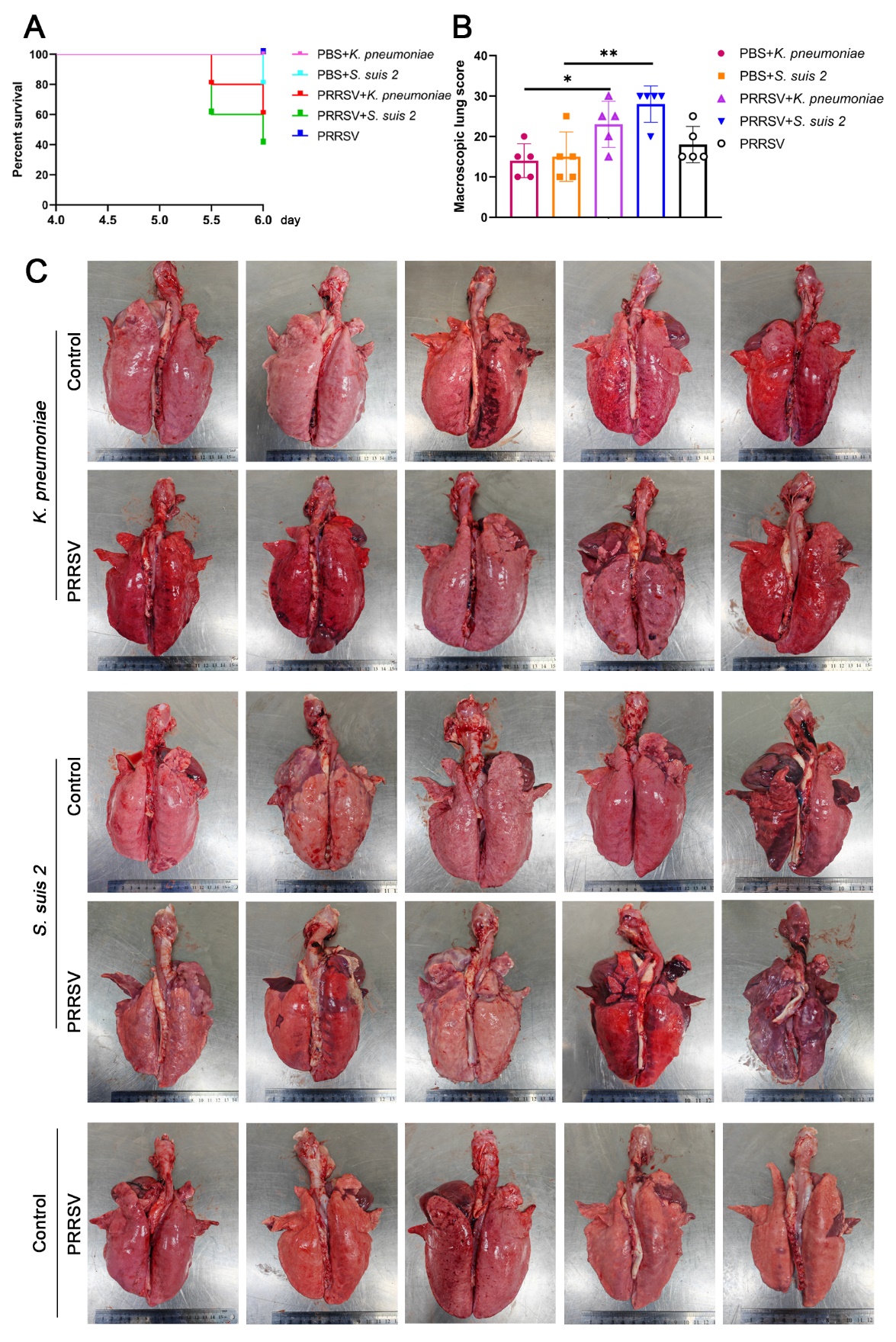


**Supplementary Figure 1. Piglet survival curve and macroscopic lung lesions of piglets**

(A) Survival curve of piglets after bacterial infection. (B) The gross lung lesion scores of piglets in each group. (C) Piglets were infected with PRRSV, when the body temperature returned to normal, piglets were infected with bacteria, and piglets were sacrificed 24 hours later, and the lung was taken to observe the macroscopic lung lesion changes.


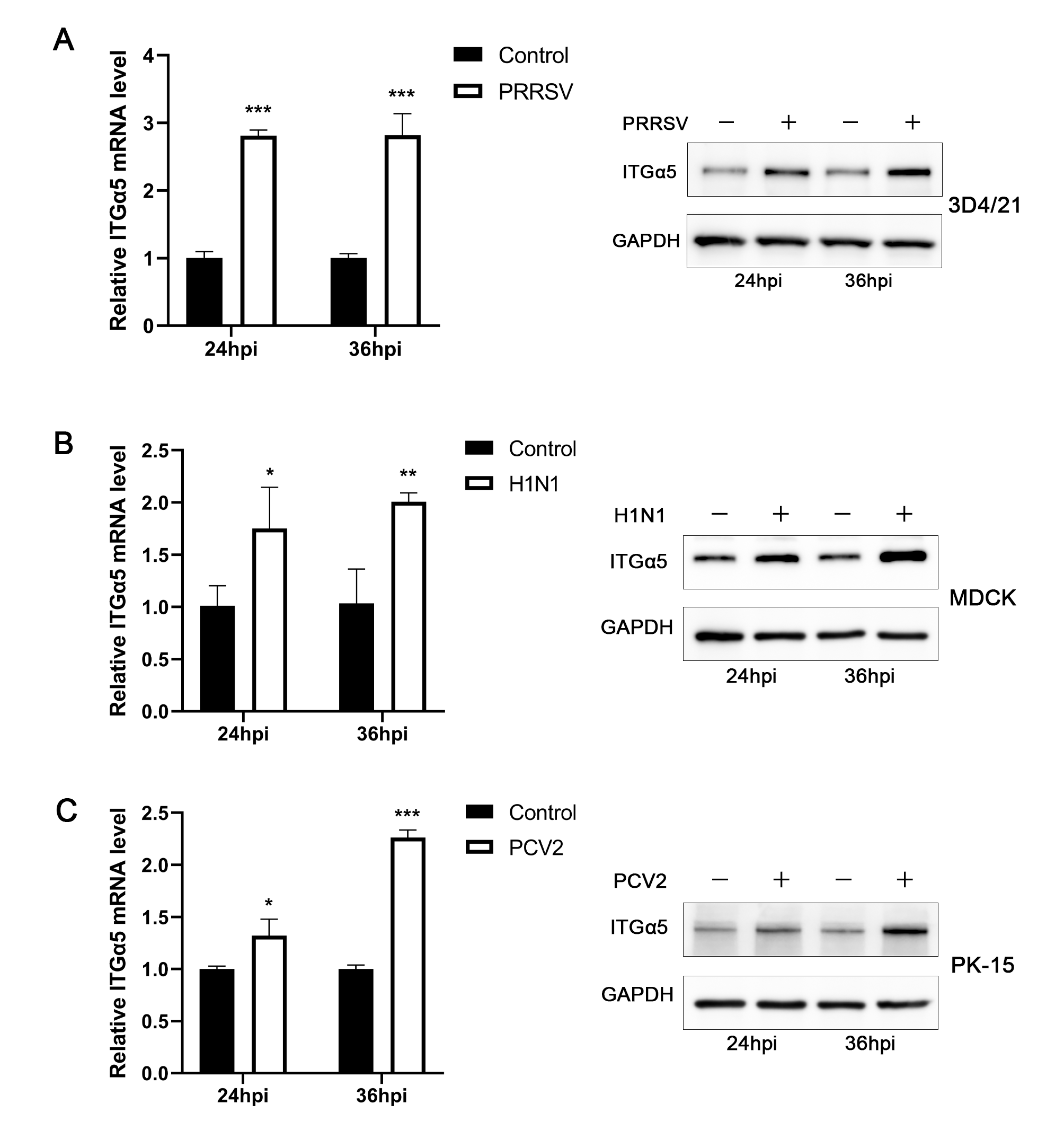


**Supplementary Figure 2.** **Viral infection promoted the ITGα5 expression**

(A) 3D4/21 cells were infected with PRRSV GD-HD (MOI=1), then the expression of ITGα5 was detected at 24 and 36 hpi. (B) MDCK cells were infected with H1N1 (MOI=1), then the expression of ITGα5 was detected at 24 and 36 hpi. (C) PK-15 cells were infected with PCV2 (MOI=1), then the expression of ITGα5 was detected at 24 and 36 hpi.


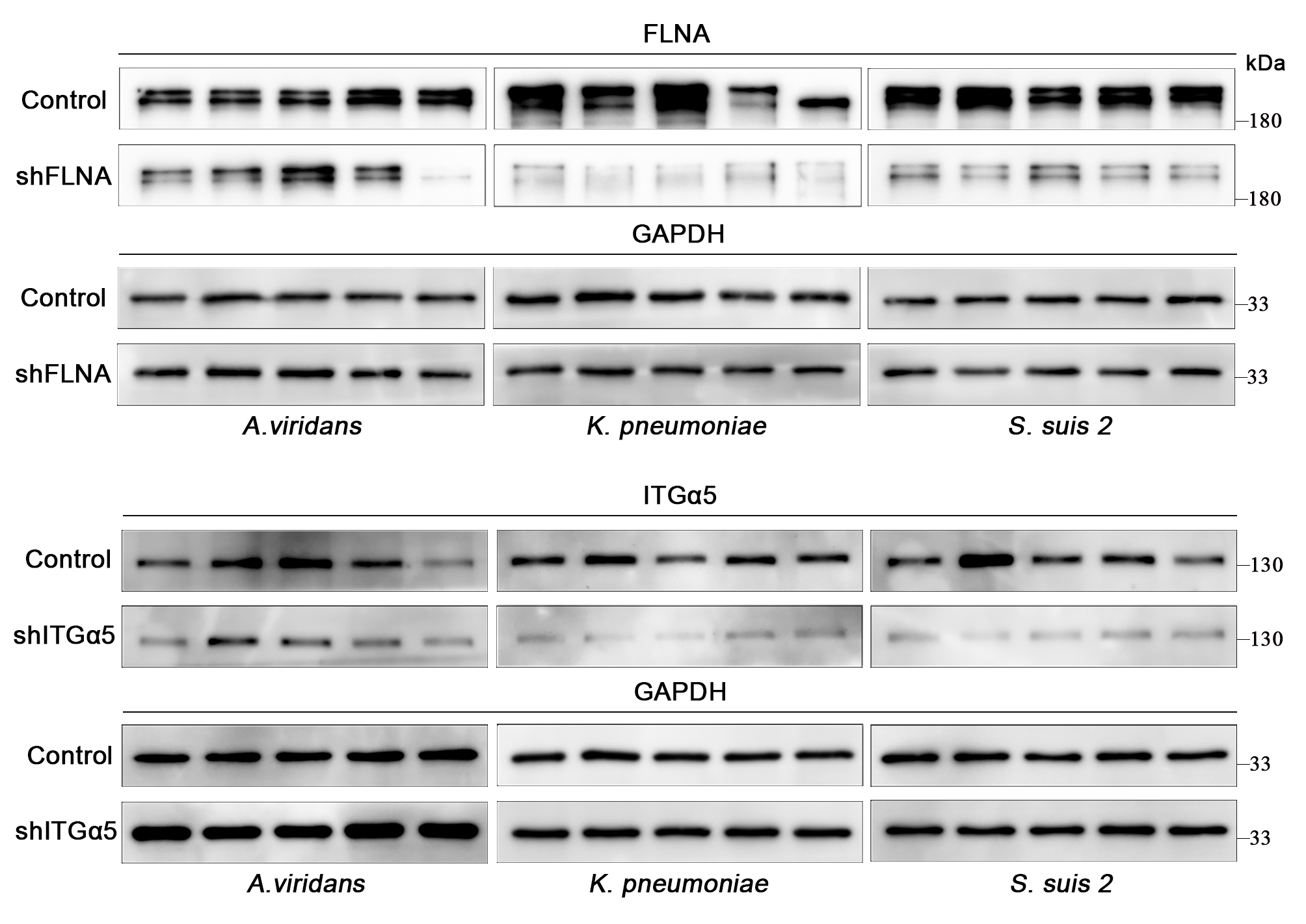


**Supplementary Figure 3. The expression levels of FLNA and ITGα5 in the lungs of lentiviral shRNA knockdown mice**

The mice were infected with shRNAs lentivirus, infected with bacteria 14 days later, and sacrificed 24 hours after bacterial infection. After the lung was homogenized, the protein of the samples was extracted, quantified by a BCA kit, and the samples were prepared for Western blot analysis.
